# Implications of physics, chemistry and biology for dosimetry calculations using theranostic pairs

**DOI:** 10.7150/thno.62851

**Published:** 2022-01-01

**Authors:** Cassandra Miller, Julie Rousseau, Caterina F. Ramogida, Anna Celler, Arman Rahmim, Carlos F. Uribe

**Affiliations:** 1 Department of Integrative Oncology, BC Cancer Research Institute, Vancouver, BC, Canada.; 2 Department of Physics and Astronomy, University of British Columbia, Vancouver, BC, Canada.; 3 Department of Molecular Oncology, BC Cancer Research Centre, Vancouver, BC, Canada.; 4 Department of Chemistry, Simon Fraser University, Burnaby, BC, Canada.; 5 Life Sciences Division, TRIUMF, Vancouver, BC, Canada.; 6 Department of Radiology, University of British Columbia, Vancouver, BC, Canada.; 7 PET Functional Imaging, BC Cancer Research Centre, Vancouver, BC, Canada.

**Keywords:** radiopharmaceutical therapy, theranostics, dosimetry, personalized medicine, radiopharmaceuticals

## Abstract

Theranostics is an emerging paradigm that combines imaging and therapy in order to personalize patient treatment. In nuclear medicine, this is achieved by using radiopharmaceuticals that target identical molecular targets for both imaging (using emitted gamma rays) and radiopharmaceutical therapy (using emitted beta, alpha or Auger-electron particles) for the treatment of various diseases, such as cancer. If the therapeutic radiopharmaceutical cannot be imaged quantitatively, a “theranostic pair” imaging surrogate can be used to predict the absorbed radiation doses from the therapeutic radiopharmaceutical. However, theranostic dosimetry assumes that the pharmacokinetics and biodistributions of both radiopharmaceuticals in the pair are identical or very similar, an assumption that still requires further validation for many theranostic pairs. In this review, we consider both same-element and different-element theranostic pairs and attempt to determine if factors exist which may cause inaccurate dose extrapolations in theranostic dosimetry, either intrinsic (e.g. chemical differences) or extrinsic (e.g. injecting different amounts of each radiopharmaceutical) to the radiopharmaceuticals. We discuss the basis behind theranostic dosimetry and present common theranostic pairs and their therapeutic applications in oncology. We investigate general factors that could create alterations in the behavior of the radiopharmaceuticals or the quantitative accuracy of imaging them. Finally, we attempt to determine if there is evidence showing some specific pairs as suitable for theranostic dosimetry. We show that there are a variety of intrinsic and extrinsic factors which can significantly alter the behavior among pairs of radiopharmaceuticals, even if they belong to the same chemical element. More research is needed to determine the impact of these factors on theranostic dosimetry estimates and on patient outcomes, and how to correctly account for them.

## 1. Introduction

Theranostics is a paradigm in which therapy and diagnosis are combined. In nuclear medicine, this is achieved by using radiopharmaceuticals that bind to the same molecular targets for both imaging and therapy 1. Pharmaceuticals labeled with either a positron- or gamma-emitting radionuclide can be imaged via positron emission tomography (PET) or single photon emission computed tomography (SPECT), respectively. In oncology, this allows for non-invasive quantification of *in vivo* tumor cell target expression. A therapeutic counterpart or “theranostic pair” radiopharmaceutical with a beta, alpha, or Auger electron emitting radionuclide may also be administered to deliver lethal radiation to the same regions of high uptake observed in the imaging procedure (e.g. tumors/metastases) 2. This type of therapy has been referred to by different names, such as molecular radiotherapy, radionuclide therapy, or targeted radionuclide therapy, and has specific special cases such as radioligand therapy (targeting receptors), peptide receptor radionuclide therapy (targeting specific kinds of receptors activated by peptides), and radioimmunotherapy (using monoclonal antibodies (mAbs)). We will broadly refer to all of these as radiopharmaceutical therapy (RPT).

**Figure 1:**
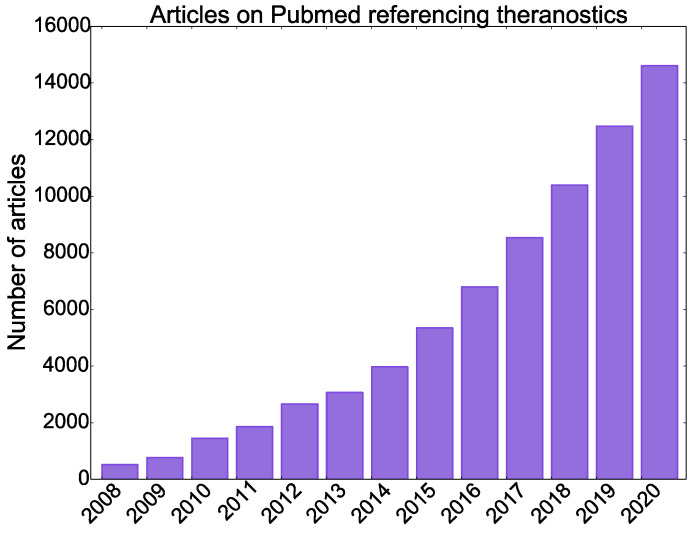
Number of articles published on PubMed containing the words “theranostics” or “theragnostics” from 2006 to 2020.

Theranostics emerged with the use of iodine-131 (^131^I) for both the diagnosis and treatment of thyroid diseases. Recently, theranostics has formed a substantial research and industry paradigm with an extensive range of applications under development 3. Figure 1 shows the exponential growth in the number of articles published on PubMed containing the phrases “theranostics” or “theragnostics” from 2008 to 2020.

Theranostics allows us to “see what we treat and treat what we see” 1. An exciting application of the theranostic concept is in patient internal dosimetry. Dosimetry is the determination of the absorbed dose (absorbed energy of ionizing radiation per unit mass) to tumors and organs-at-risk. In most RPTs, the amount of radioactivity injected in patients is either standardized or scaled by bodyweight 4 without taking into account that the absorbed dose to tumors and organs-at-risk can vary widely between patients 5-7. This “one-size-fits-all” approach might leave some patients undertreated while inducing radiation toxicity for some others 8. Dosimetry allows the optimization of the injected amount of the radiopharmaceutical which enables treatment planning prior to new cycles of therapy and thus personalization of treatment.

There are many challenges which currently prevent dosimetry from widespread clinical use. It is costly and complex, and guidelines for optimal image reconstruction or analysis remain to be developed and/or widely adopted 9,10. Tumor segmentation in particular is challenging, as patients may present with multiple metastatic lesions of various sizes, some of which are not visible; there are many proposed segmentation methods 11,12 but none have been widely adopted. Dosimetry may also be a burden on some patients, who may be asked to return for up to three or four SPECT or PET scans per cycle. In any case, there are significant ongoing efforts towards simplified dosimetry protocols 13 and analysis workflows 14,15.

However, there is growing evidence that personalized treatment can improve patient outcomes. Correlations between absorbed dose and treatment response for certain cancers have been established 16-22 although conclusive evidence of a dose-response relationship is limited and more research is required. Clinical trials such as the Dosisphere trial 18 are needed and should be encouraged.

Quantitative images, which are required for dosimetry, are difficult to obtain for some therapeutic radionuclides. For example, yttrium-90 (^90^Y) can only be quantified by its Bremsstrahlung emissions or very low positron emission. Actinium-225 (^225^Ac) could potentially be imaged due to its gamma emitting daughters (francium-221 (^221^Fr) and bismuth-213 (^213^Bi)), but due to the injected activities being very low, it is challenging to obtain good counting statistics in a clinically acceptable scan duration.

To overcome this, imaging nuclides such as indium-111 (^111^In) and zirconium-89 (^89^Zr) have been used as surrogates to predict therapeutic absorbed doses or to perform retrospective dosimetry after therapy 23-25. We call this use of an imaging surrogate “theranostic dosimetry”. The imaging and therapeutic nuclides together are called a “theranostic pair”. The term “theranostic” can refer to either a pharmaceutical labeled with a radioisotope that can be imaged and used for therapy at the same time (e.g. lutetium-177 (^177^Lu)), or to a pharmaceutical that can be labeled with one radioisotope for imaging (e.g. gallium-68 (^68^Ga)) and with another for treatment (e.g. ^225^Ac). In this work we primarily use it to refer to a theranostic pair of radioisotopes, but we also discuss ^177^Lu in detail due to its importance in the field. Theranostic dosimetry with the use of an imaging surrogate can be performed using two different isotopes or chemical elements to label a given pharmaceutical. The validity of this approach has, to our knowledge, not been fully established, and needs to be validated for each theranostic pair.

With the promising results observed in RPT for cancers such as that of the neuroendocrine system 26 and prostate 27, in addition to the goal of making personalized absorbed dose assessments a reality, there is an increasing need to understand if dosimetry calculations with theranostic pairs are accurate. In the present work, we discuss the validity of performing dosimetry with theranostic pairs and try to answer the question: *is it possible to accurately determine absorbed dose estimates for a therapeutic radiopharmaceutical by extrapolation from an imaging counterpart, when different isotopes or elements are used to label a given pharmaceutical?*

We first explain theranostic dosimetry in detail and discuss sources that may lead to inaccurate dosimetry estimates. We present a non-inclusive list of pharmaceuticals along with theranostic radionuclides they are commonly labeled with and their targeted diseases, with a focus on oncology. We review and discuss intrinsic and extrinsic factors relating to the (i) chemistry of the radiopharmaceuticals, (ii) biological behavior in the body, and (iii) physics of the radionuclides that can alter *in vivo* behavior in the body or lead to incorrect dosimetry estimates. We also analyze a few theranostic pairs in more detail and attempt to gather evidence indicating whether these pairs are suitable for theranostic dosimetry. Two types of theranostic pairs will primarily be investigated in this work: (i) same-element (e.g. ^86^Y/^90^Y) same-pharmaceutical pairs and (ii) different-element (e.g. ^111^In/^90^Y) same-pharmaceutical pairs.

We note that the most common use of theranostic imaging is in identifying patients suitable for RPT and assessing the treatment efficacy. Here, we focus on gathering understanding regarding the accuracy of theranostic dosimetry.

## 2. Theranostic Dosimetry

The committee on Medical Internal Radiation Dose (MIRD) has proposed a schema using quantitative imaging to estimate radiation absorbed doses in RPT 9,28,29. The most common method is dosimetry at the organ-level, which requires obtaining quantitative images at different time points after the injection of the therapeutic radiopharmaceutical to measure its biodistribution and pharmacokinetics. Quantitative images are used to generate time activity curves (TACs) for each region/volume of interest (ROI/VOI), e.g. lesion or organ-at-risk. The total number of emissions in a particular ROI or VOI is given by the time integrated activity (TIA) which is the integral of the TAC. The TIA is multiplied by the “S-factor” (also called the “dose factor”) to obtain the dose within the ROI/VOI. The S-factor represents the absorbed dose in tissue for one decay of the therapeutic radionuclide and contains information about the energy of the different emissions of the radionuclide and the fraction of energy deposited in a target region; it is dependent on the radionuclide and the absorption characteristics of the tissue of interest. S-factors have been developed by groups such as RADAR and can be precalculated at the organ- 30 or voxel-level 31 with the former typically being done for an average patient anatomy, and the latter allowing for a more personalized approach 32.

While organ-level dosimetry only provides the mean absorbed dose in the tissue of interest, voxelized dosimetry allows one to obtain a 3D absorbed dose distribution. Voxel-level dosimetry may use voxelized S-values or dose point kernels, which are scoring matrices of the radial absorbed dose distributions around a voxel or point source, respectively, of a given radioisotope, usually placed in water 32. Usually created with Monte Carlo (MC) simulations, the dose point kernel is convolved with a patient's TIA distribution (obtained from PET or SPECT scans) to obtain a voxelized absorbed dose map.

The current gold standard for dosimetry is the full MC method, which uses MC simulations of photon and particle transport within anatomically realistic phantoms (derived from patient computed tomography (CT) images), combined with the activity distribution from patient-specific PET or SPECT images, to obtain voxelized absorbed dose estimates in tissues of interest 31. While this method is the most accurate, it is very computationally intensive and time consuming. Alternative approaches to full MC simulations are also being actively explored 33.

In theranostic dosimetry, the surrogate's TAC (which includes information about the biological pharmacokinetics of the radiopharmaceutical), is decay corrected using the physical half-lives of the imaging and therapeutic radionuclides to predict the TAC of the therapeutic radiopharmaceutical. Theranostic dosimetry estimates are not only useful to prevent toxicity but can also be used to determine optimal activities for subsequent therapy cycles.

The popular radionuclide ^177^Lu is theranostic on its own as it can be imaged quantitatively with SPECT and the beta particles from its decay are useful for treatment. Regardless, an imaging surrogate may still be beneficial for pre-therapy dosimetry for nuclides like ^177^Lu. In this context, “pre-therapy dosimetry” refers to dosimetry done prior to therapy, either to determine an optimal injected activity for therapy, or to predict the therapeutic absorbed doses.

Two primary assumptions are made when utilizing the imaging surrogate approach for dosimetry:

(i) The radiopharmaceuticals have similar biodistributions (i.e. the degree of accumulation in different organs in the body at a given timepoint) even if variables such as the injected amount of each radiopharmaceutical, the physiology of the patient, or the expression level of the target pre, intra, or post-therapy differ.

(ii) Both radiopharmaceuticals have similar pharmacokinetics (i.e. rates of absorption, distribution, metabolism, and elimination) in the tissues of interest.

**Figure 2:**
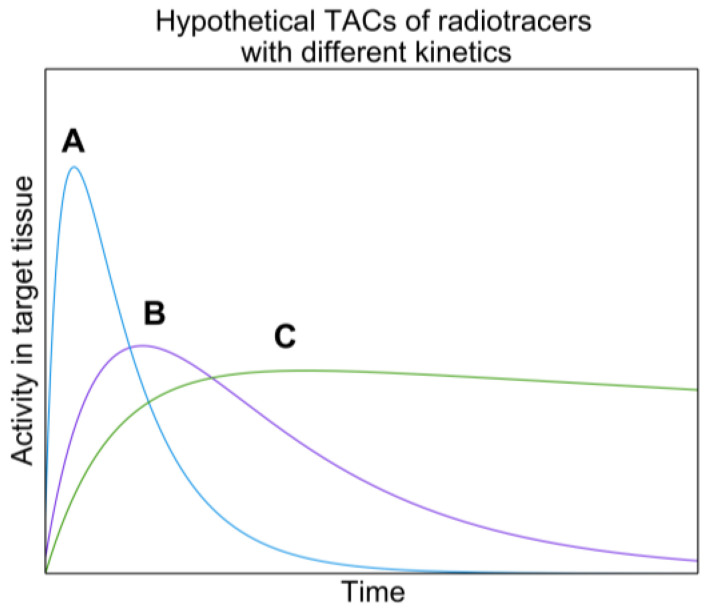
Bi-exponential TACs of hypothetical radiopharmaceuticals with varying uptake and washout kinetics in a tissue of interest. Curves represent radiopharmaceuticals with **A:** fast uptake and clearance, **B:** intermediate uptake and slow clearance, **C:** slow uptake and long retention. The total number of disintegrations (i.e. TIAs) will be quite different for each of these curves resulting in different absorbed dose values.

If these two assumptions are not met, the extrapolated TAC from the surrogate will not match the therapeutic radiopharmaceutical's TAC and incorrect absorbed doses will be obtained. Figure 2 shows hypothetical TACs of radiopharmaceuticals with different kinetics. One can imagine how integrating these TACs will result in significantly different TIAs.

While the TACs generally differ when the imaging and therapeutic radiopharmaceuticals are different biological targeting vectors, it is often assumed that switching a therapeutic radioisotope/nuclide to an imaging one using the same tracer backbone does not alter the biological behavior of the radiopharmaceutical in any way. However, this is not guaranteed to be true. It is therefore important to assess the validity of this statement for various theranostic pairs and to understand the uncertainties in absorbed dose prediction with this approach.

## 3. Classes of Theranostic Pairs

Several components of a radiopharmaceutical can be changed: the chemical element, the isotope of the given element, the pharmaceutical, and/or the chelator (a molecule that ensures the stable complexation of the radionuclide to the pharmaceutical (such as with radiometals)). In this work, we primarily discuss changing only the element or isotope. We focus on 3 different classes of theranostic pairs:

1) “*Same-element isotope pairs*” in which the imaging and therapeutic radioisotopes belong to the same chemical element and they are both used to label the same chemical compound. Tables 1 and 2 show examples of medical uses and decay information for some of these pairs. For example, [^86/90^Y]Y-DOTATOC 34 for neuroendocrine tumors (NETs) and [^124/131^I]I-Metaiodobenzylguanidine (mIBG) for neuroblastomas 35.

Isotopes have the same number of electrons and protons, the only difference being the number of neutrons. The chemical and biological behavior of an element is mainly determined by its electronic structure, and so it is assumed that isotopes of the same chemical element have identical intrinsic chemical behavior 36. However, extrinsic factors exist that can alter the biological behavior, which are discussed in Sections 4 and 5.

2) “*Different-element pairs*” in which the imaging and therapeutic radionuclides belong to different chemical elements and are both used to label the same chemical compound. Tables 1 and 2 show examples of medical uses and decay information for some of these pairs. For example, copper-64 (^64^Cu) labeled DOTATATE ([^64^Cu]Cu-DOTATATE) and [^177^Lu]Lu-DOTATATE for NETs 37. Different elements used to label a pharmaceutical are not guaranteed to have identical *in vivo* biological behavior in the body, but it is often assumed they do for treatment planning and dosimetry.

3) “*Different-pharmaceutical pairs*” in which different radiopharmaceuticals that target the same molecule are labeled with radioisotopes or radionuclides of the same or different elements. For example, [^68^Ga]Ga-PSMA-11 (PSMA being prostate specific membrane antigen) and [^177^Lu]Lu-PSMA-617 38 for prostate cancer. This class of pairs will not be discussed much besides a select few, such as PSMA-617/PSMA-11 and technicium-99m (^99m^Tc) labeled macroaggregated albumin (MAA)/^90^Y microspheres.

## 4. Factors That Can Affect the Biological Behavior of Radiopharmaceuticals

The biological behavior of a radiopharmaceutical is a critical component for subsequent dosimetry estimates. Before we look at this in more detail, we want to highlight a few points:

(i) Estimated absorbed dose calculations, even when not following the imaging surrogate approach but using the therapeutic radiopharmaceutical itself, may already only be accurate within 10-20% in the best case scenario 103. If the additional inaccuracy introduced by the use of an imaging surrogate is small, the impact on the estimated absorbed dose may be marginal.

(ii) Some effects discussed in the following may require more investigation to be fully understood. As such, it may be difficult to isolate the true causes of some of these phenomena. For instance, if the pharmacokinetics of a radiopharmaceutical is altered post-therapy, we may not know if this is due to damage at a cellular level, a difference in injected amounts pre- vs. post-therapy, the saturation of receptors, or any other possible effect. Furthermore, most of the time, if not all, it is likely a combination of many factors that impact the biological behavior of a radiopharmaceutical.

(iii) If the difference in behavior between two radiopharmaceuticals is known precisely, we could potentially correct for it, e.g. by applying a scaling factor to account for differences in biodistributions between the imaging and therapeutic radiopharmaceutical. Therefore, while this review aims to uncover behavioral differences between radiopharmaceutical pairs, the presence of differences does not necessarily mean that an imaging surrogate should not be used or that dosimetry estimates are guaranteed to be inaccurate. More research is required to determine the precise impact of behavioral differences on dosimetry estimates.

(iv) Ultimately, the most important goal of dosimetry is to improve patient outcomes. If pre- or post-therapy dosimetry with an imaging surrogate improves patient outcomes compared to no dosimetry or only dosimetry with the therapeutic radiopharmaceutical, theranostic dosimetry should still be performed even if large differences in biological behavior between the radiopharmaceuticals are discovered, as long as toxicity is not a possible side effect.

Below, we discuss general factors that can cause differences in the biodistributions and/or the pharmacokinetic behavior of radiopharmaceuticals. We include both intrinsic (e.g. chemical properties) and extrinsic (e.g. injected amount) factors that may result in those differences.

### 4.1 Injected Amount/Mass of the Radiopharmaceutical

The biological behavior of a radiopharmaceutical can change via the so-called “mass effect”, which causes a change in uptake in certain organs depending on the injected mass or amount (e.g. the molar amount or the activity). This effect is dependent on the binding site. Easily saturable sites such as cell surface-bound PSMA receptors can only accommodate a finite number of bound ligands; the uptake will not change regardless of how much more radiopharmaceutical is injected 104. On the contrary, non-saturable and intermediately saturable sites, such as bone and changes in glucose transporter, do not seem to be affected by the injected mass of, for example, fluorine-18 (^18^F) labeled sodium fluoride (NaF) (a bone seeking agent) and [^18^F]FDG (fluorodeoxyglucose, a glucose analogue) respectively 105.

This effect is well understood for certain pharmaceuticals; for example NHL patients treated with ibritumomab tiuxetan (also called Zevalin, an anti-CD20 murine IgG1 κ mAb for NHL) are injected with unlabeled rituximab before therapy to block the CD-20+ binding sites on B-cells in non-target organs, enabling better accumulation of Zevalin on tumor cells 106.

**Figure 3:**
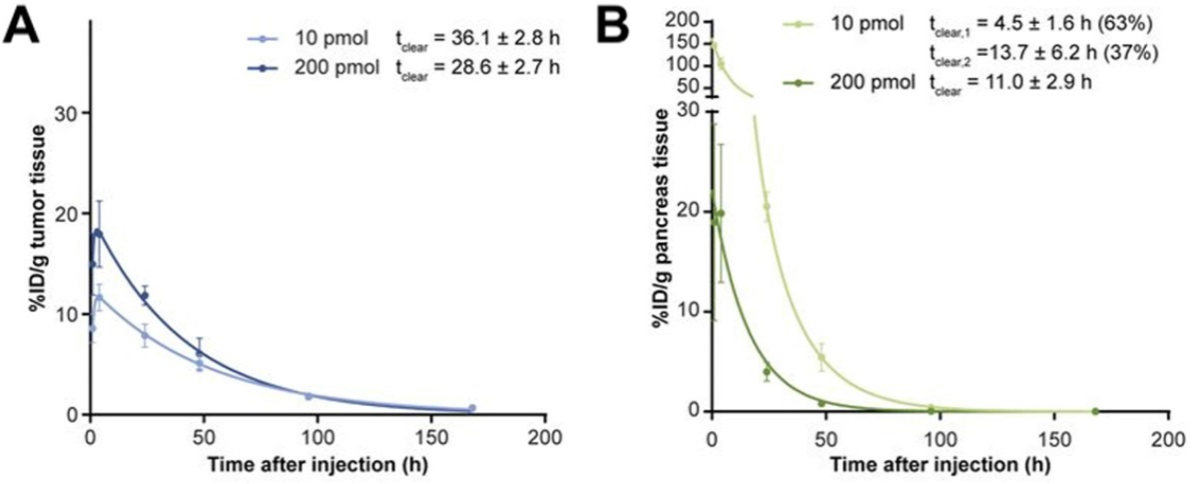
TACs of [^177^Lu]Lu-NeoBOMB1 in the tumor (**A**) and the pancreas (**B**) of mice (N = 4 per time point). Adapted with permission from 66, copyright 2017, SNMMI.

A pre-clinical study in which mice were injected with 200 picomoles of [^177^Lu]Lu-NeoBOMB1 (a GRPR antagonist) resulted in a 1.3 times higher tumor absorbed dose and a 5 times lower pancreas absorbed dose compared to an injection of 10 picomoles. The tumor and pancreas clearance half-lives of [^177^Lu]Lu-NeoBOMB1 also changed (Figure 3) 66. A decrease in uptake in non-target organs was also found with [^177^Lu]Lu-OPS201 (an SSTR2 agonist) and [^111^In]In-J591 (an anti-PSMA antibody) with increasing injected mass 98,107. Increasing the mass of [^111^In]In-J591 from 10 mg to 100 mg increased the residence time in the whole body, serum, and tumor lesions of patients by 10 minutes to 29 hours (depending on the region), and decreased it in the liver by 11 hours 98.

**Figure 4:**
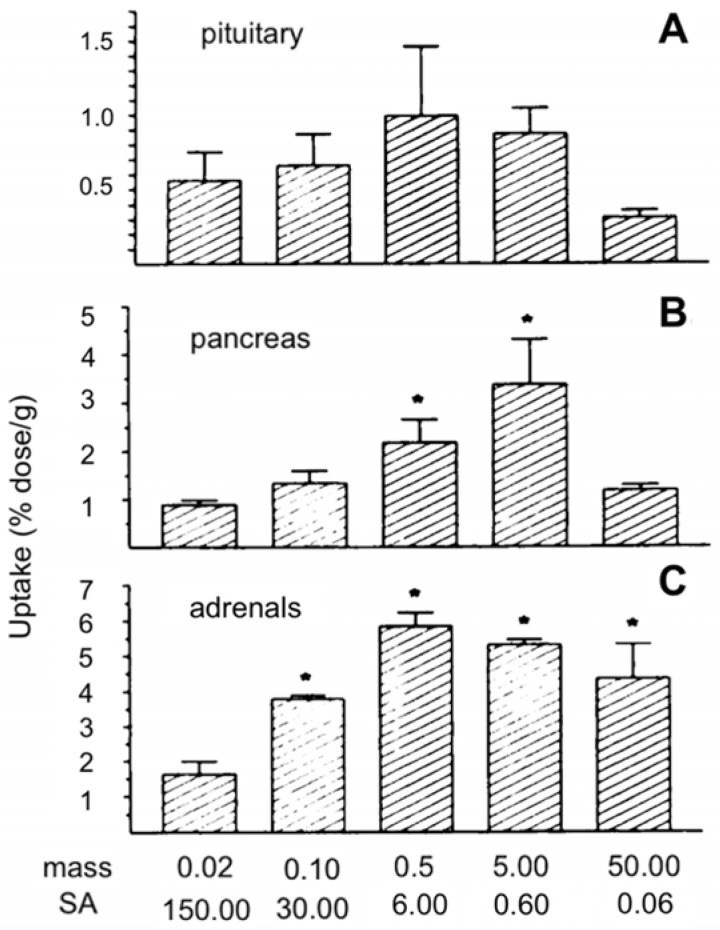
The %ID/g of [^111^In]In-pentetreotide in the pituitary glands (**A**), pancreas (**B**), and adrenals (**C**) of rats 24 hours after injection of varying masses of pentetreotide (µg) (N = 3). The activity of ^111^In was 3 MBq at each mass. *p < 0.05 (significantly different from 0.02 µg). Adapted with permission from 108, copyright 1995, SNMMI.

Increasing the injected mass of pentetreotide (a SSTR2 agonist) bound to a constant activity of ^111^In increased the percentage injected dose per gram (%ID/g) in SSTR-positive organs of mice, shown in Figure 4, but did not impact the %ID/g in SSTR-negative organs 108.

These effects are likely due to the tumor and target organs expressing significantly more receptors than the non-target tissues. A higher target-to-background ratio is beneficial for visualization and treatment purposes but may create issues for theranostic dosimetry. The injected amount of the imaging radiopharmaceutical is almost always much lower than its therapeutic counterpart. For example, the standard injected activity in a therapeutic cycle of [^177^Lu]Lu-DOTATATE is 7.4 GBq 8, but less than 200 MBq of [^68^Ga]Ga-DOTATATE is used for imaging 109. Based on approximate specific activities (SAs) of [^177^Lu]Lu- and [^68^Ga]Ga-DOTATATE 109,110, these activities correspond to 235 nmol and 15.6 nmol of ^177^Lu- and ^68^Ga-labeled DOTATATE respectively, a difference of > 15-fold.

#### Impact on theranostic dosimetry

Injecting different radiopharmaceutical amounts/masses may change the target-to-background ratio and the residence time in certain tissues. This primarily occurs when the binding sites are saturable and may be less important with non-saturable and intermediately saturable sites. If not accounted for during theranostic dosimetry, this effect could result in inaccurate dose estimates. This effect likely differs on a patient-by-patient basis; it remains to be seen whether methods with personalized correction factors can be developed to correct for this. In any case, if population-based correction factors were determined for each theranostic pair, it may render theranostic dosimetry estimates more accurate.

### 4.2 Large Differences in Physical Half-Life

Dosimetry requires capturing the rate of accumulation, retention, and elimination of the radiopharmaceutical in the tissue of interest. Even if the pharmacokinetics of the imaging and therapeutic radiopharmaceuticals are identical, the imaging radiopharmaceutical may be unable to capture the behavior at later time points if its physical half-life is too short compared to its biological half-life (i.e. we will not be able to image at those time points).

Statistics decrease as the imaging radionuclide decays, and image quality may become poor, potentially leading to less reliable quantitative data and inaccurate TACs (though the extent of this depends on scanner sensitivities and performances). The radionuclide may even decay before the radiopharmaceutical has finished its retention or elimination stage in tissue. For example, [^177^Lu]Lu-PSMA-617 has been shown to have an effective half-life of 33.0 hours in the kidneys 111, while the physical half-life of ^68^Ga is only 68 minutes. An effective half-life of 33.0 hours corresponds to 11.6 half-lives of ^68^Ga, at which point sequential quantitative imaging is likely not able to capture the biological half-life of PSMA-617.

Furthermore, the uptake of a radiopharmaceutical is not instantaneous. It first circulates in the blood, the rate of uptake in tissues depending on factors such as blood flow rate, degree of binding to blood components, and permeability across capillaries 112. The shorter the imaging surrogate's physical half-life, the sooner images must be taken; depending on the isotope and the pharmaceutical the image may only capture the blood clearance phase prior to accumulating in the tissue of interest. This occurs with [^123^I]I-mIBG (13.2 hour physical half-life) and [^131^I]I-mIBG (8.02 day physical half-life) 43.

As discussed in Section 2, one could decay correct the imaging radiopharmaceutical's TAC to attempt to obtain the therapeutic radiopharmaceutical's TAC 42. The imaging radiopharmaceutical's TAC is used to estimate its biological half-life, which is then combined with the physical half-life of the therapeutic radioisotope/nuclide to provide an extrapolated estimate of the TAC in the therapeutic cycle:



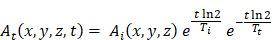



where 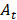 and 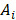 are the activities of the therapeutic and imaging radiopharmaceuticals at point x, y, z at time t respectively, and 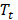
and 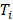 are the physical half-lives of the therapeutic and imaging radionuclides respectively. This leads to a more accurate dosimetry estimate. However, if the biological half-life of the radiopharmaceutical is long and the physical half-life of the imaging surrogate is short, the predicted washout kinetics may be incorrect. According to MIRD Pamphlet No. 16, ignoring the long-term slow washout component of the TAC can result in errors in the residence time calculation of up to 45% 29. Different methods of extrapolating the washout curve after the last imaging time point have been used, assuming, for example, purely physical decay, or using a fit of the pre-existing TAC to extrapolate the rest of the curve 42.

#### Impact on theranostic dosimetry

If the physical half-life of the imaging radionuclide is much shorter than that of the therapeutic radionuclide, it might decay before the pharmacokinetics needed to replicate the therapeutic radiopharmaceutical are completely captured. This would result in an incorrectly predicted TIA which would lead to an incorrect absorbed dose estimate. If the true washout curve of the therapeutic radiopharmaceutical is unknown, it may be impossible to correct for this.

### 4.3 Chemical Differences

Chemical differences between radiopharmaceuticals play a large role in determining their biological behavior. This is primarily an issue between different-element and different-pharmaceutical pairs, as same-element isotope pairs are assumed to have the same intrinsic chemical behavior 36. In this section, we will primarily discuss factors that impact radiometals, as most of the radionuclides discussed in this work are as such.

**Figure 5:**
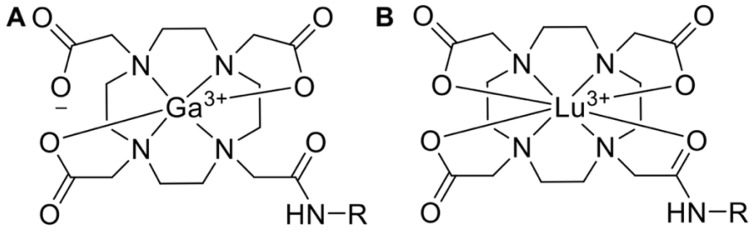
^68^Ga (**A**) and ^177^Lu (**B**) complexed to DOTA, both forming overall neutral complexes, but in different geometries (in this example DOTA has been conjugated to a targeting vector, R, via one of the carboxymethyl arms).

Pharmaceuticals labeled with a radiometal (such as ^177^Lu, ^68^Ga, ^90^Y, ^225^Ac, etc.) are called coordination compounds. The coordination complexation chemistry of the radiometal into the chelator will vary depending on the radiometal. For example, ^68^Ga^3+^ forms a hexadentate (N_4_O_2_) complex with DOTA in octahedral geometry, where two carboxylate groups remain free 113. On the other hand, the ^177^Lu^3+^ ion forms an octadentate complex with DOTA, which means there are no free carboxylate groups, shown in Figure 5.

Various aspects of a radiopharmaceutical's coordination chemistry can impact its biological behavior in different ways. For example, increasing the positive charge of the chelator binding a ^64^Cu-labeled antibody increased its kidney uptake by 6-fold, presumably because the increased positive charge increased the interaction with negatively charged kidney basal cells 114. The structure and charge distribution of [^68^Ga]Ga-/[^111^In]In-DOTA is different although they have identical charges in their complex, resulting in ^111^In-labeled conjugates showing more rapid blood clearance than those labeled with ^68^Ga, potentially because the charge distribution influences transient binding to blood proteins 115.

Changing the radionuclide used to label a pharmaceutical can alter factors like the lipophilicity or hydrophilicity of the final compound. Lipophilic compounds may diffuse more freely into tissues and can undergo intracellular biotransformation or binding to the tissue leading to longer retention 112, which can impact the binding of the pharmaceutical to the receptor site 116, while radiopharmaceuticals with lower lipophilicity tend to be excreted more rapidly via the urinary system 117. In addition, differences in pH, ionic strength, or osmolality between two radiopharmaceuticals can alter the *in vitro* stability, which impacts the pharmacokinetics 118.

If the radiopharmaceutical is unstable, the radionuclide may become uncomplexed in the body and roam freely. This can be caused by demetallation (the breaking of the bond between a metal and a molecule), radiolysis (the dissociation of molecules caused by the radiation itself) or by the enzymatic degradation of peptides. For example, demetallation of ^89^Zr from [^89^Zr]Zr-DFO-mAb conjugates (DFO being deferoxamine, a commonly used chelator for ^89^Zr) can result in high liver uptake in mice 119, and would also be expected in bone 120. A case study showed radiolysis of [^68^Ga]Ga-PSMA resulted in increased vascular activity in the patient 121.

There are also radiopharmaceutical dependent chemical differences that need to be considered. While the FDA approved [^177^Lu]Lu-DOTATATE, Lutathera, is conjugated with DOTA, excess DTPA is added to chelate any free ^177^Lu ions that remain in the mixture as they accumulate in bone and can result in significant absorbed dose 122,123. The choice of chelator alters the biodistribution: 4 hours after rats were injected with [^177^Lu]Lu-DOTATATE or [^177^Lu]Lu-DTPA, the total body retention was 20% or 4% of the injected amount respectively 123. The change in biodistribution due to the addition of DTPA would likely not be caught by an imaging surrogate.

#### Impact on theranostic dosimetry

Pharmaceuticals labeled with different radiometals (or chelators) can have chemical differences that result in different biodistributions, pharmacokinetics, and/or molecular stabilities. This could introduce errors in theranostic absorbed dose estimates with different-element pairs. Similar to the mass effect, this effect likely differs on a patient-by-patient basis, although population-based correction factors may be able to improve accuracy of theranostic dosimetry estimates.

### 4.4 Changes in Pharmacokinetics after Therapy

The pharmacokinetics of a radiopharmaceutical in a patient may change intra- or post-therapy because of various biological effects, i.e. the loss of tumor cells after therapy or the formation of antibodies against the tracer, which has been observed in some patients treated with octreotide and rituximab 124-126. This effect causes the radiopharmaceutical to bind to the antibodies instead of targeted tissues. In a case study of a patient treated with [^111^ln]In-octreotide, there was a 10-fold higher binding of labeled octreotide in blood serum 20 months after therapy due to the presence of octreotide antibodies 124. Furthermore, anti-rituximab antibodies were detected in 23% of patients treated with rituximab 126.

Treatment of NETs with [^90^Y]Y-DOTATOC doubled the residence time of [^111^In]In-octreotide in many organs nine weeks post-therapy, significantly increasing the biological effective dose (BED) (Table 4). If post-treatment dosimetry was not performed, the maximum-tolerable red marrow absorbed dose of 2 Gy would have been exceeded in subsequent therapy cycles. The same study found a similar effect with [^68^Ga]Ga-DOTATOC PET/CT, in which a decrease in spleen uptake and an increase in the blood pool retention of the tracer was seen 7 weeks after therapy, with the SUV_mean_ dropping from approximately 180 to approximately 5 in the spleen. In this case, no antibodies against the tracer were found 96.

Altered behavior of a radiopharmaceutical post-therapy is most important when using dosimetry to determine optimal injected activities for subsequent cycles of therapy. With each cycle of therapy, the initial dosimetry estimate may become less accurate as pharmacokinetic changes occur. While pre-therapy dosimetry may enable more accurate prediction of dosage for the first therapy cycle, these results highlight the importance of reassessing dosimetry after each cycle to track pharmacokinetic changes and the importance of personalized dosimetry in general. Each patient is likely to respond differently to therapy and may or may not reach absorbed dose limits to organs-at-risk. Without personalized dosimetry, there is no way to assess these differences. We do note that this effect is also important outside of dosimetry with an imaging surrogate, when using the therapeutic radiopharmaceutical to determine absorbed doses after the first therapy cycle.

#### Impact on theranostic dosimetry

The pharmacokinetics and/or biodistribution of the radiopharmaceutical can change in between cycles due to effects such as the creation of antibodies against the tracer. This can result in over/undertreatment if the dosage is based on dosimetry prior to or after the first therapy cycle only. At this time, it is unclear how to account for this effect because it likely varies wildly amongst patients. The imaging surrogate is also subject to post-therapy pharmacokinetic changes, which highlights the importance of performing dosimetry after each cycle.

### 4.5 Specific Activity and Presence of Chemical Impurities

The specific and molar activities of a material (e.g. a radionuclide or a radiopharmaceutical) are defined as the radioactivity per mass (Bq/g) and per mole (Bq/mol) respectively. Depending on how the radionuclide and radiopharmaceutical are manufactured, they may contain traces of the stable radionuclide (known as the carrier) and/or other chemical impurities. Only some radionuclides are truly carrier-free, and they are still susceptible to contamination when the final radiopharmaceutical is being synthesized. The SA of a radiopharmaceutical is usually reported, although it is not considered during dosimetry estimates.

Lowering the specific or molar activity of a radioisotope/nuclide decreases its incorporation into the pharmaceutical during radiolabeling. Radiolabeling is performed by direct labeling methods or by the use of a chelator 127, both which may lead to a lower SA of the final radiopharmaceutical. For example, the chelator may bind to other non-radioactive metal impurities in the labeling solution which can be present in concentrations several orders of magnitude greater than the radiometal itself 127. If there are a limited number of receptor sites on tissue cells, the non-labeled molecules will compete with the radiolabeled ones, which could change the target-to-background ratio 118. The SA is important because if the imaging radiopharmaceutical has a different target-to-background ratio than its therapeutic counterpart due to differing SAs, errors in theranostic dosimetry estimates may occur.

Decreasing the SA of [^68^Ga]Ga-DP11 (a PSMA inhibitor) by 16-fold did not significantly change the uptake in the tumors of mice, but resulted in a 6-fold lower renal uptake 128. No-carrier added [^131^I]I-mIBG had a 1.25 to 2 times lower uptake in organs such as the adrenals, liver, kidneys, thyroid, bone, etc. at certain timepoints compared to 148-185 MBq/mg SA [^131^I]I-mIBG, while there were no significant difference in tumor uptake 129. An increase in target-to-background ratio was also found when increasing the SA of [^111^In]In-pentetreotide 108.

Chemical impurities reduce the radiopharmaceutical's radiochemical purity, which is the percentage of the stated radionuclide in the final radiopharmaceutical. Impurities may be formed because of the nuclear reaction used to produce the radionuclide or later on during the labeling process because of unwanted chemical reactions/radiolysis 118. Chemical impurities can cause the radiopharmaceutical to localize in different areas of the body than expected 118. Impurities in the form of other radioactive isotopes can also contribute to the patient absorbed dose. For instance, if 0.2% of a sample of ^123^I is replaced by ^125^I, the ^125^I alone will add 12% to the thyroid absorbed dose 130.

The impact of differing SAs and presence of chemical impurities impact same-element and different-element pairs equally, as both different elements and isotopes of the same element are synthesized differently and therefore are expected to contain different amounts of impurities and have different SAs.

#### Impact on theranostic dosimetry

Altering the SA of a radiopharmaceutical can change its biodistribution and target-to-background ratio. This effect depends on the tissue and the binding site. Similarly, chemical impurities can alter the biodistribution or result in dose over/underestimation. Both of these effects can impact theranostic dosimetry estimates. The impact of differences in SA on dosimetry estimates may be able to be somewhat accounted for using a population-based correction factor, although this would not catch individual variations among patients. It is unclear how to account for chemical impurities as they depend on the supplier and how the radionuclide/pharmaceutical was synthesized.

### 4.6 Patient Behavior

Patient behaviors such as diet, exercise, smoking/drug use, and the use of certain medications can all alter a radiopharmaceutical's biodistribution and/or kinetics 118,131,132. For example, a low-carbohydrate diet compared to a high-fat/low-carbohydrate diet prior to [^18^F]FDG scans reduced the mean myocardial SUV_max_ by 1.9 times 131. Antidepressants and other drugs can reduce the tumor uptake of [^123^I]I-mIBG 132, and previous cancer treatments can change the biodistribution of a radiopharmaceutical 118.

Patient behavior can impact dosimetry in different ways depending on the length of time between the administration of the imaging and therapeutic radiopharmaceuticals. To our knowledge, for most therapies there are no prescribed guidelines for how long to wait between administering each radiopharmaceutical, but ranges such as 8 days apart (for mIBG 35) and 7-9 days apart (for Zevalin 133) have been suggested. Ranges are target dependent; when imaging/treating targets that are easy saturated, the imaging and therapy radiopharmaceuticals may need to be administered at separate times to ensure that maximal receptors are available. The target turnover is important to consider in this context.

Although not exactly a patient behavior, dosimetry estimates may be impacted by concomitant medications given during therapy such as amino acid infusions to reduce renal uptake. Amino acids and other positively charged molecules can reduce renal doses by 20-55% 134. Infusions are sometimes given during pre-therapy scans with an imaging surrogate 135, but not always 47; the infusions can cause severe nausea and vomiting 135 and would rather be avoided.

It is difficult and maybe impossible to ensure that the radiopharmaceutical biokinetics will be identical for the imaging and therapy scans. Patients may have gained or lost weight, started or stopped medications, etc. The position of the patient may also differ between each scan, which makes image registration difficult; however methods have been proposed to account for this 136,137. The impact of this on theranostic dosimetry is difficult to account for and depends on many factors, such as the radiopharmaceutical used, the disease it is used to treat, and the patients themselves. It is important to note that this issue is not confined to theranostic dosimetry; changes in patient behavior could impact the quantitative accuracy of multi-time point scans of any isotope. Regardless, it should still be considered as a potential source of error during dosimetry.

#### Impact on theranostic dosimetry

The biological behavior of a radiopharmaceutical in a given patient may change on a day-to-day basis due to changes in diet, drug/medication use, etc. If imaging and therapy are performed at different times, differences in the biodistributions or radiopharmaceutical kinetics could impact dosimetry estimates. These differences can be reduced by administering the imaging surrogate as close to the therapeutic injection as possible, but this is not always possible due to factors like receptor saturation. Obvious behavioral changes in factors like diet, smoking, or drug use should attempt to be minimized in between imaging and therapy.

## 5. Examples of Theranostic Pairs

We will now discuss a few of the theranostic pairs listed in Tables 2 and 3 in more detail. Specifically, we investigate if there is evidence showing biodistribution or pharmacokinetic differences between the radiopharmaceuticals in the pair. We also analyze other factors that could impact dosimetry, such as issues related to accurate imaging of the radiopharmaceuticals. Overall, we attempt to validate if each pair is suitable for theranostic dosimetry.

Before this, we would like to discuss the ideal scenario, which is that the therapeutic radionuclide can be imaged quantitatively during therapy. Currently, the most popular nuclide for this is ^177^Lu, a beta emitter that has shown excellent therapeutic efficacy with DOTATATE 138, PSMA-617 139, and others. ^177^Lu also emits gamma photons and can be imaged with SPECT. The ability of ^177^Lu to be imaged quantitatively and be used therapeutically allows it to be a theranostic powerhouse.

**Figure 6:**
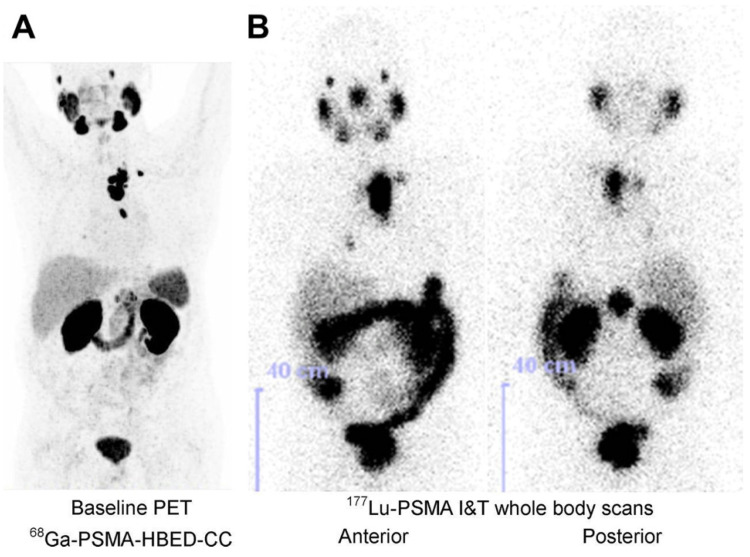
Maximum-intensity projection of [^68^Ga]Ga-PSMA-HBED-CC PET/CT, taken 60 minutes post-injection of 164 MBq (**A**). [^177^Lu]Lu-PSMA I&T SPECT image, taken 47 hours post-injection of 5.7 GBq (**B**). Adapted with permission from 65, copyright 2015, SNMMI.

Even in this near ideal scenario, an imaging surrogate for ^177^Lu is still beneficial for pre-therapy dosimetry. For pre-therapy imaging, a lower half-life nuclide is preferable to reduce absorbed dose (the half-life of ^177^Lu is 6.6 days, while the half-lives of the common PET imaging nuclides ^68^Ga and ^18^F are 68 and 109 minutes respectively). The higher sensitivity of PET also allows the acquisition of higher quality images (Figure 6) 65.

Therefore, although some of the therapeutic radionuclides discussed below can be used for dosimetry without a surrogate, finding an accurate surrogate is beneficial for pre-therapy dosimetry as it may enable dosimetry without any additional scans being performed. Table 5 indicates the theranostic pairs that will be discussed in the following sections and how the physical half-lives of the imaging surrogates compare to their therapeutic counterparts, which plays a significant role in the quantitative accuracy of dosimetry as discussed in Section 4.2.

### 5.1 ^124^I, ^123^I and ^131^I

One of the oldest examples of same-element isotope pairs is ^123^I or ^124^I for SPECT or PET respectively and ^131^I for therapy. Free ^131^I has been in use for decades in the treatment and diagnosis of thyroid diseases, as it emits both betas for therapy and gammas for SPECT imaging. The thyroid has a natural uptake of iodine, so iodine itself can be used without an additional carrier molecule. Radiopharmaceuticals labeled with ^131^I and its imaging surrogates are listed in Table 2. Dosimetry using ^131^I itself has been performed 140, however less than 10% of ^131^I's decay products are suitable for gamma imaging, which leads to poor quality images.

[^123^I]I-mIBG has been shown to underestimate the absorbed dose from [^131^I]I-mIBG compared to using a combination of pre-therapy [^123^I]I-mIBG and intra-therapy [^131^I]I-mIBG scans 43 (intra-therapy scans referring to scans with the therapeutic injection). This is due to ^123^I's short physical half-life (13.2 hours) compared to ^131^I (8.02 days). The TAC of [^123^I]I-mIBG may only capture the blood clearance phase of the radiopharmaceutical, while images of [^131^I]I-mIBG can capture the longer retention in the body that is more relevant for dosimetry 43.

Pre-therapy [^123^I]I-mIBG dosimetry and intra-therapy [^131^I]I-mIBG dosimetry found a low correlation between organ absorbed doses, with a coefficient *r* value of 0.30 for the heart wall and liver, 0.60 for the lung, and 0.52 for the total body (note that these values may also be impacted by post-therapy pharmacokinetic changes, discussed in Section 4.4) 44. Regardless, the absorbed doses calculated with [^123^I]I-mIBG were more conservative than those with [^131^I]I-mIBG, which could result in undertreatment if post-therapy dosimetry is not performed 44.

^124^I is preferred over ^123^I for diagnostic imaging because it can be imaged with PET, yielding higher sensitivity, resolution, and image quality, and it has a half-life of 4.2 days which is beneficial for dosimetry as it allows tracking later stages of the pharmacokinetic curve. Theranostic dosimetry with ^124^I has been performed for pharmaceuticals such as mIBG 35, MIP-1095 (a PSMA inhibitor) 45, and NaI (for thyroid uptake) 42. To the best of our knowledge, these results have not been compared to similar absorbed dose estimates with the ^131^I-labeled counterpart.

**Figure 7:**
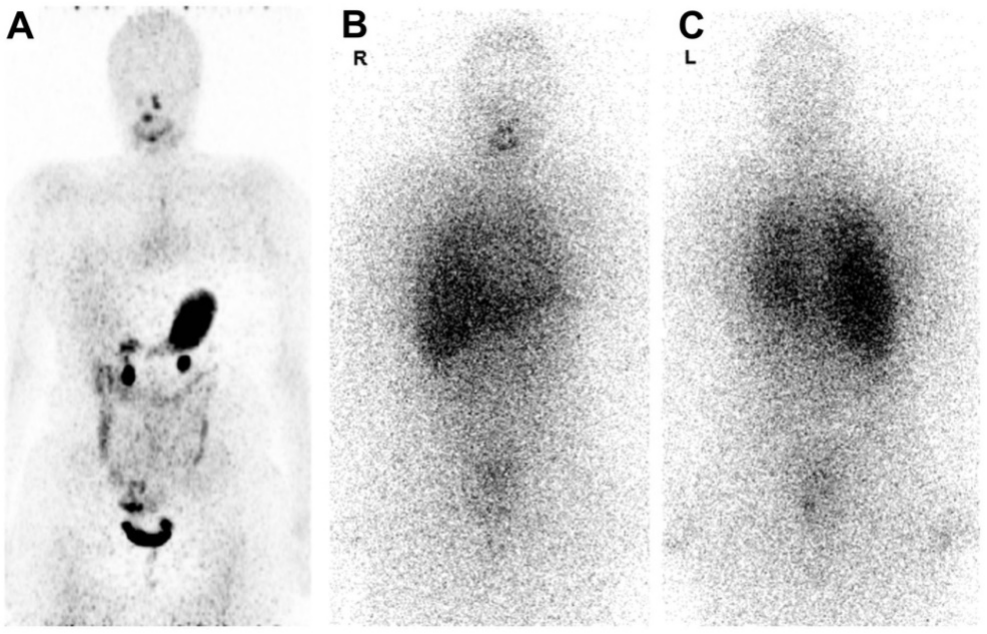
^124^I PET/CT image taken 1-4 days post-injection of 74 MBq (**A**). ^131^I WBS images taken 1 week post-injection of 5.5-7.4 GBq (anterior (**B**), posterior (**C**)). Adapted with permission from 141, copyright 2016, SNMMI.

Figure 7 shows ^124^I PET/CT and ^131^I whole-body scintigraphy (WBS) images in the same patient 141. ^124^I PET/CT images are less noisy but they show no pathologic uptake, while the ^131^I WBS shows disseminated lung metastases 141. Image noise can reduce quantification accuracy, however, relying on ^124^I scans alone may have resulted in a false negative in this particular case. This study found that out of 13 ^124^I PET/CT scans, 5 were false-negatives, which the authors believe may be caused by both technical and biological factors, such as the amount of iodine in the patient's diet 141.

**Figure 8:**
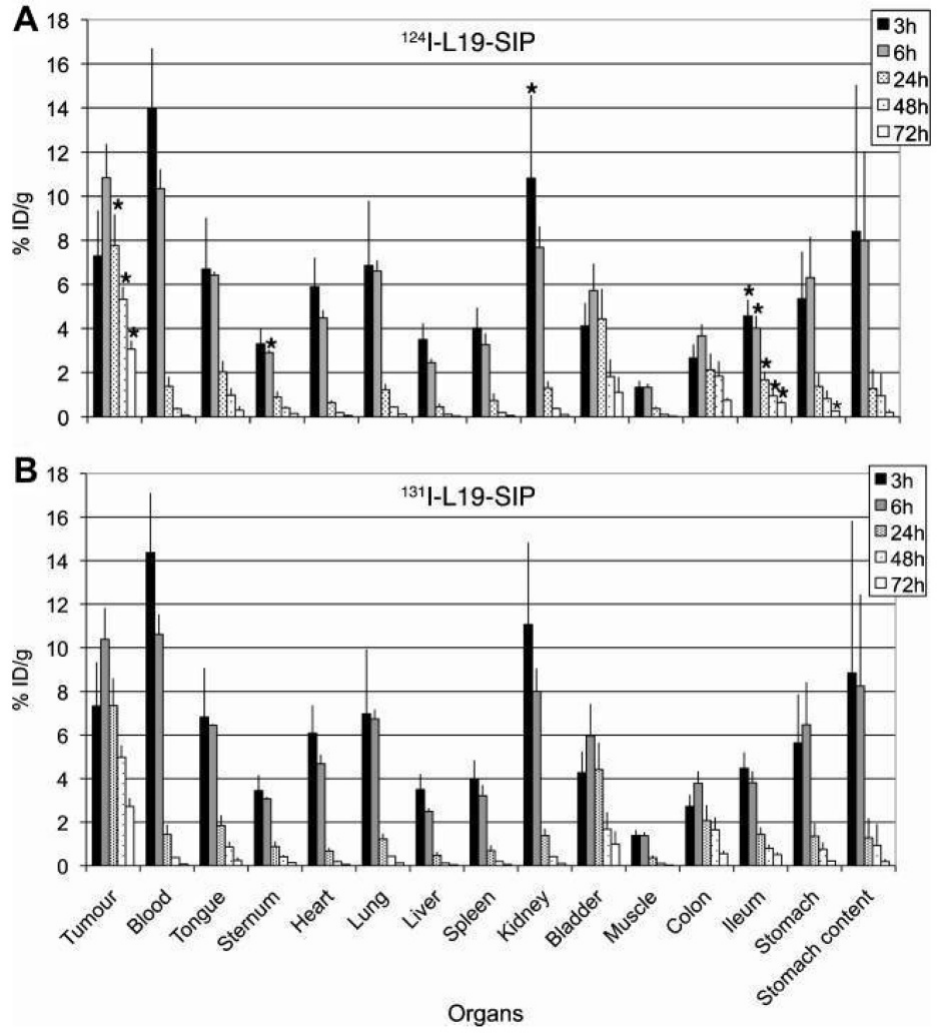
Biodistributions of 0.37 MBq of [^124^I]I-L19-SIP (**A**) and [^131^I]I-L19-SIP (**B**) in tumor-bearing nude mice (N = 4/group). Statistically significant differences in uptake are marked with an asterisk in plot A. Adapted with permission from 46, copyright 2009, EJNMMI.

The biodistributions of [^124^I]I- and [^131^I]I-L19-SIP (a mAb targeting the extra domain B of fibronectin) have been compared in preclinical studies with mice (Figure 8). The biodistributions are nearly identical in most organs, although there are statistically significant differences in uptake in the ileum, kidney, sternum, and tumor 46.

Regardless of the choice of iodine isotope, dosimetry errors may arise after thyroid imaging or therapy because of “thyroid stunning”, which is the decreased uptake of radioiodine in thyroid tissue after the initial diagnostic scan. This phenomenon may be due to the destruction of thyroid cells from radiation or from the reduced ability of thyroid cells to uptake radioiodine for a specific time interval after the first injection 142. This effect occurs with both ^131^I and ^123^I 143,144, but is thought to be less common with ^124^I, potentially due to the lower required activity 145.

Thyroid stunning may result in an overestimated thyroid absorbed dose if pre-therapy dosimetry is performed, as the therapeutic uptake is lower after the stunning occurs. This effect is difficult to account for; Hilditch et al. showed that the impact of stunning is incredibly variable among patients - when ^131^I was used for intra-therapy imaging, the intra-therapy to pre-therapy uptake ratio ranged from 6-93% and 17-130% when ^131^I and ^123^I was used for pre-therapy imaging, respectively 144.

Post-therapy behavioral changes of the radiopharmaceutical have also been observed when treating with mIBG (which is almost exclusively labeled with radioiodine). Fielding et al. performed dosimetry with [^131^I]I-mIBG 1-3 weeks prior to therapy, and then again immediately post-therapy, finding a lower tumor uptake. However, there was also a mean increase of 290% of carrier mIBG reported for the therapeutic administration compared to pre-therapy, so the altered behavior could be due to post-therapy effects, increase in the amount of carrier mIBG, or both 146.

#### Suitable for theranostic dosimetry?

Previous studies suggest that ^123^I is not suitable for theranostic dosimetry because its short half-life results in failing to capture the true TAC of ^131^I, at least when used to label mIBG. ^124^I appears to be a more suitable imaging surrogate for ^131^I, although may sometimes result in false negatives. Both isotopes could cause thyroid stunning which could lead to thyroid absorbed dose overestimation if not considered.

### 5.2 ^99m^Tc,^ 111^In, ^86^Y and ^90^Y

^90^Y, a pure beta emitter, is one of the most frequently used radionuclides for cancer therapies (see Tables 2 and 3). Imaging ^90^Y can be done with PET or via Bremsstrahlung imaging with SPECT. However, image quantification is challenging with both modalities because the positron yield is very low, and because of the continuous nature of the Bremsstrahlung spectrum 147,148. Because of the numerous FDA-approved tracers labeled with ^90^Y, an imaging surrogate for this nuclide would be incredibly advantageous. Here we discuss ^99m^Tc,^ 111^In and ^86^Y, three commonly used imaging surrogates for ^90^Y; the first two can be imaged with SPECT, and the third with PET.

[^99m^Tc]Tc-MAA SPECT imaging is routinely performed before radioembolization with ^90^Y microspheres (usually for treatment of hepatocellular carcinoma), for various purposes such as dosimetry, determining the percent shunting to the lungs 101, aiding in patient selection, determining optimal injected activities for therapy, and more. The mechanism of this pair is different than other examples in this work as microspheres are not pharmaceuticals per se; instead they are injected directly into the hepatic artery which feeds liver tumor tissue.

Studies show large variations in agreement between absorbed doses/activity distributions between [^99m^Tc]Tc-MAA and ^90^Y SPECT/PET (in both tumors and healthy tissue), ranging from good correlations to poor 101,102,149,150, with one study finding a difference of > 10% in the activity distribution of 68% of all analyzed VOIs with [^99m^Tc]Tc-MAA compared to ^90^Y SPECT 151.

Studies that found good correlations often still reported outliers in which significant discrepancies between pre- and post-therapy dosimetry were found 101,102. This is likely because the biodistributions are impacted by multiple factors that may be difficult to control, such as reproducibility of the administration procedure (e.g. the catheter tip position 101), differences in physical properties between MAA particles and ^90^Y microspheres, differences in hepatic or tumor vascularization 102, and variations in particle sizes, injection techniques, and flow rates between ^90^Y microspheres and MAA particles 149. There may be up to 300 times more microspheres injected compared to MAA particles, which could impact activity concentrations 152.

Many of these issues are difficult to avoid, and at this time dosimetry may not be as accurate if based on the MAA distribution. Correlations with biological response, which may be the most important factor, have also been conflicting, with some studies showing treatment response does not correlate with [^99m^Tc]Tc-MAA uptake 152,153, and some showing positive correlations between pre-therapy tumor absorbed dose and treatment response 154.

While pre-therapy imaging with [^99m^Tc]Tc-MAA is required regardless to determine the percentage of lung shunting, the activity concentration of ^90^Y is higher in this treatment compared to other RPTs with ^90^Y due to the direct injection into the hepatic artery. Therefore, dosimetry with ^90^Y may be more accurate during radioembolization than in other RPTs in which the concentrations are lower.

^111^In has been used for theranostic dosimetry of ^90^Y with pharmaceuticals such as Zevalin 23, J591 98, and DOTATATE 95. Significant pharmacokinetic and chemical differences have been observed between pharmaceuticals labeled with these two radionuclides.

Firstly,^ 111^In-labeled DOTA-biomolecule conjugates are more hydrophilic and more fluxional (non-rigid) than the same conjugates labeled with ^90^Y at room temperature 155. ^90^Y may also accumulate more in the bone marrow than ^111^In, resulting in differences in the absorbed bone marrow dose 99,100; the distribution within the bone marrow also differs when labeled to pharmaceuticals such as the mAb B3 100. The bone marrow is often a dose-limiting organ, especially for long-lived radiopharmaceuticals such as antibodies; a study found that using ^111^In for dosimetry could result in a bone marrow dose 1.7 times higher than expected 99. Considering that the maximum bone marrow dose is only 2 Gy, this could lead to toxicity.

Albeit similar blood plasma pharmacokinetics between the ^90^Y- and ^111^In-labeled mAbs, two studies have confirmed a faster and slower urinary excretion of ^90^Y-labeled mAbs at early and late time points, respectively 99,100. Whole body clearance times of ^111^In- and ^90^Y-labeled antiTac (a murine IgG2a mAb targeting the IL-2Rα receptor for T-cell leukemia) were found to be 314 and 450 hours, respectively - a difference of 136 hours 99.

Theranostic dosimetry using ^86^Y has been performed for pharmaceuticals such as DOTATOC 34,40, trastuzumab (an anti-HER2/neu mAb for ovarian carcinomas) 40, and PSMA targeting compounds 41. ^86^Y has a shorter half-life (14.7 hours) compared to ^90^Y (2.7 days), which may result in late time-point scans of ^86^Y failing to accurately model that of ^90^Y 156. There is limited research comparing pharmacokinetic differences between ^86^Y- and ^90^Y-labeled pharmaceuticals, although we assume this pair is subject to the same external factors discussed in Section 4 of this work. An additional factor that may affect theranostic dosimetry is challenges in quantitative imaging of ^86^Y.

^86^Y positron decay is accompanied by at least 1-3 simultaneous gamma emissions (with energies of 1077 or 1854 keV, and additionally a 628 and/or 443 keV emission in 33 and 17% of all decays respectively 157), leading to multiple prompt coincidences. Prompt coincidences involve two photons being emitted simultaneously that are accepted by the PET scanner (with each other or with an annihilation photon). They cannot be distinguished from a true coincidence event, or be entirely corrected for with the usual techniques used to correct for random coincidences 158. ^86^Y also decays via electron capture, which contributes to degrading PET images 157, and has a wide and high energy gamma spectrum (470 keV to 3880 keV), that can be accepted within the PET acquisition window directly or after scattering, resulting in decreased resolution and inaccuracies in dead time correction 159.

The reduction in quantitative accuracy due to prompt gammas is more severe for ^86^Y than other non-pure positron emitters such as ^124^I, causing activity (and therefore absorbed dose) overestimation 160. These effects can result in an activity overestimation of 90% if not corrected for 161. However, [^86^Y]Y-DOTATOC has still been used to obtain dose-effect relationships for [^90^Y]Y-DOTATOC therapy 19,20, and so this surrogate is still promising despite these potential imaging limitations.

#### Suitable for theranostic dosimetry?

[^99m^Tc]Tc-MAA may be a poor surrogate for ^90^Y microspheres, due to the nature of the radioembolization administration procedure and the differences between MAA and glass/resin microspheres. ^90^Y may be a better choice for liver and tumor dosimetry during radioembolization therapies, due to the higher activity concentrations. ^111^In also does not seem to be a good imaging surrogate for ^90^Y because of the chemical and biological differences between yttrium and indium labeled pharmaceuticals. Despite the possible PET imaging issues and differences in physical half-life, ^86^Y is likely a better imaging surrogate for ^90^Y. Because of the challenges of imaging ^90^Y, performing dosimetry with ^86^Y is likely more accurate than with ^90^Y at this time, however this still requires more study.

### 5.3 ^68^Ga, ^44^Sc, ^64^Cu, ^18^F, ^89^Zr, and ^177^Lu

As discussed above, although ^177^Lu can be used for dosimetry during therapy, an imaging surrogate would be beneficial for pre-therapy dosimetry. Here we will discuss the use of ^68^Ga, scandium-44 (^44^Sc),^ 64^Cu, ^18^F, and ^89^Zr as imaging surrogates for ^177^Lu, all of which are imaged with PET. Pharmaceuticals commonly labeled with ^177^Lu and its surrogates can be found in Table 3.

The theranostic pair ^68^Ga and ^177^Lu is currently one of the most popular different-element pairs being investigated for theranostic purposes 66,162. As discussed briefly in Section 4.2, ^68^Ga is unlikely to be able to capture the full shape of the washout curve of ^177^Lu and might not be suitable for late time-point imaging. The different coordination chemistry between ^68^Ga and ^177^Lu also results in differing lipophilicities, hydrophilicities, charge, receptor affinities and pharmacokinetics between labeled pharmaceuticals 67,163-165.

**Figure 9:**
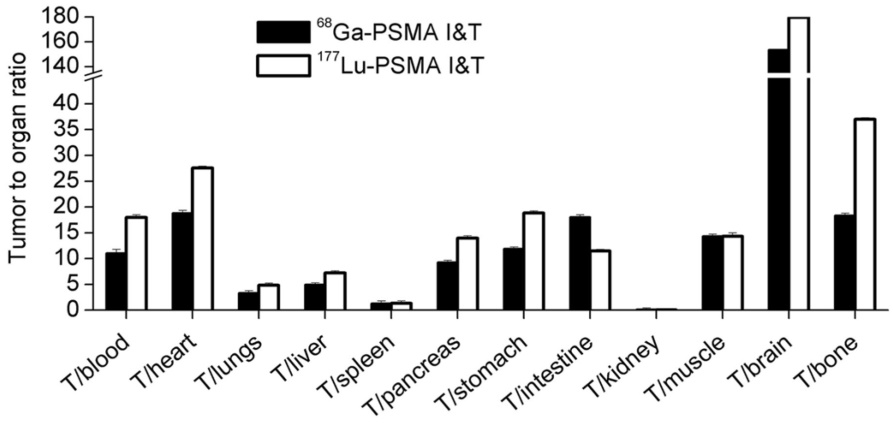
Tumor-to-organ ratios (%ID/g) of [^68^Ga]Ga- and [^177^Lu]Lu-PSMA I&T in mice, 1 hour post-injection (N = 4/group). Adapted with permission from 46, copyright 2015, SNMMI.

The %ID/g of [^177^Lu]Lu-PSMA I&T was found to be higher than [^68^Ga]Ga-PSMA I&T in certain tissues of tumor-bearing mice, such as the tumors, kidneys, and the spleen 65. The tumor-to-organ ratios are shown in Figure 9. Similarly, [^68^Ga]Ga- and [^177^Lu]Lu-DOTA^ZOL^ (a bone-targeting pharmaceutical) had the same skeletal retention in mice, but [^177^Lu]Lu-DOTA^ZOL^ had a faster blood clearance and an approximately 7 times higher kidney uptake than [^68^Ga]Ga-DOTA^ZOL^ at 1 hour post-injection 70.

Several clinical studies have attempted to correlate standard uptake values (SUVs) from ^68^Ga PET scans with absorbed doses calculated using therapeutic ^177^Lu SPECT images. The results of some of these studies are presented in Table 6. All of these studies determined at least a moderate correlation between ^68^Ga PET/CT and ^177^Lu SPECT/CT scans, which is promising. [^44^Sc]Sc-PSMA-617 has been used for pre-therapy dosimetry of [^177^Lu]Lu-PSMA-617 in patients 83,84, and has been shown to have more similar *in vivo* kinetics to [^177^Lu]Lu-PSMA-617 than [^68^Ga]Ga-PSMA-11 85, likely due to having more similar coordination chemistry 164,165. [^44^Sc]Sc-PSMA-617 and [^177^Lu]Lu-PSMA-617 were found to have nearly identical hydrophilic properties, binding affinities to PSMA, and a similar tissue distribution profile in mice 85. More research is needed to compare the biodistributions and pharmacokinetics of these radiopharmaceuticals.

[^64^Cu]Cu-DOTATATE (also called Detectnet) was recently FDA approved in the US, allowing it to be used clinically for imaging NETs. [^64^Cu]Cu-DOTATATE was found to detect more NET lesions in patients than [^68^Ga]Ga-DOTATATE 166, and combined with the longer half-life may be preferable for pre-therapy dosimetry.

The biological behavior of [^177^Lu]Lu- and [^67^Cu]Cu-DOTA-triglycine-F(ab′)_2_ (chCE7 antibody fragments which bind to L1-CAM for various cancers) have been compared in tumor-bearing mice, and the %ID/g of the ^177^Lu-labeled conjugate was found to be twice as high in the kidneys at 24 hours post-injection compared to the ^64^Cu-labeled conjugate (34.5 %ID/g versus 16.0 %ID/g). The reduction in copper uptake was thought to be due to the negative charge of the Cu-DOTA complex compared to the neutral Lu-DOTA complex 79; this therefore may occur with any copper pharmaceutical using DOTA as a chelator (such as [^64^Cu]Cu-DOTATATE). The kidneys are a dose-limiting organ in NET therapies, and an underestimated absorbed dose could result in kidney toxicity.

Despite differences in catabolism and complexation chemistry, [^177^Lu]Lu- and [^64^Cu]Cu-cetuximab (a mAb which binds to EGRF, expressed on a number of cancers) were found to have similar uptake rate constants (the probability that the molecule will be trapped in the tissue due to antibody-antigen linking) in esophageal squamous cell carcinoma tumors, but different release rate constants (the probability the molecule will be released from the target), with coppers being higher by 5-fold 82.

^89^Zr (which has the longest half-life of the nuclides discussed in this section) may be a promising surrogate to image long-lived pharmaceuticals such as antibodies. The uptake of ^89^Zr- and ^177^Lu-labeled cetuximab in mice were found to be comparable in tumors and most other tissues, although significantly higher for ^89^Zr in the thighbone and sternum at most time points 71. We note that the chelators did differ (N-sucDf for ^89^Zr while p-SCN-Bz-DOTA and p-SCN-Bz-DTPA for ^177^Lu), which is known to impact biodistributions 114,167. At 144 hours post-injection, the mean thighbone uptake was between 4.8 and 6.9 %ID/g for [^89^Zr]Zr-N-sucDf-cetuximab, 1.0 %ID/g for [^177^Lu]Lu-p-SCN-Bz-DOTA-cetuximab, and 2.0 %ID/g for [^177^Lu]Lu-p-SCN-Bz-DTPA-cetuximab 71. Because of the bone seeking behavior of ^89^Zr 168, it is reasonable to assume this effect may be present even if identical chelators were used. This could potentially result in bone marrow absorbed dose overestimation which could result in undertreatment.

Finally, ^18^F has been proposed as a surrogate for ^177^Lu, particularly with PSMA binding tracers 169. To our knowledge, theranostic dosimetry of ^177^Lu using ^18^F has not been published. ^18^F has significant chemical differences to ^177^Lu; fluorine is not a radiometal and requires different pharmaceuticals and labeling strategies than ^177^Lu. For example, PSMA-617 cannot be labeled with ^18^F but it can be labeled with ^177^Lu. DCFPyL is a PSMA targeting molecule that can be labeled with ^18^F providing advantages for PET imaging. However, DCFPyL has a different structure than PSMA-617 170.

The use of a single pharmaceutical that can be labeled simultaneously with ^18^F and ^177^Lu may be more appropriate. Several compounds have been developed including DOTA-[^18^F]-AMBF_3_-PSMA 171. Such compounds could be labeled with ^18^F/^nat^Lu for imaging, and ^nat^F/^177^Lu for therapy. This is an exciting development as the conformation of the pharmaceutical and its properties are expected to be the same for each application. This would not bypass issues caused by half-life differences, but it would open the door to an exciting new area of research with significant potential for theranostic applications.

#### Suitable for theranostic dosimetry?

All of the imaging surrogates discussed here showed differences in biological behavior compared to ^177^Lu, with the exception of ^44^Sc, although this may be due to a lack of research comparing ^177^Lu- and ^44^Sc-labeled pharmaceuticals. ^68^Ga might not be the best choice as a surrogate for ^177^Lu, because of chemical differences and significant physical half-life differences. However, ^68^Ga has shown some correlation between PET measured SUVs and ^177^Lu therapeutic absorbed doses. ^64^Cu may underestimate absorbed doses to organs-at-risk such as the kidneys, depending on the chelator used. As a [^64^Cu]Cu-DOTA labeled pharmaceutical was recently FDA approved, theranostic dosimetry with this radionuclide may become a feasible alternative. However, more research to understand the differences between [^64^Cu]Cu-DOTA and [^177^Lu]Lu-DOTA labeled pharmaceuticals and the errors in absorbed dose predictions should be done before it is adopted into common practice. ^89^Zr is promising as at this time significant biodistribution differences have only been observed in bone, however because of this care needs to be taken when estimating bone marrow absorbed doses. Finally, new developments into pharmaceuticals which can be labeled with both ^18^F and ^177^Lu simultaneously may open the door to exciting new theranostic applications.

### 5.4 ^177^Lu, ^111^In,^ 89^Zr, and ^225^Ac

Recently, there has been great excitement for the use of alpha emitters such as ^225^Ac for RPT 172. While ^225^Ac could in principle be imaged with SPECT because of the gamma emissions of its daughters, injected activities are low to avoid toxicity which creates a challenging situation for quantitative imaging. Here, we will discuss ^177^Lu, ^111^In and ^89^Zr as potential surrogates. ^177^Lu and ^111^In can be imaged with SPECT, while ^89^Zr is a PET radionuclide.

**Figure 10:**
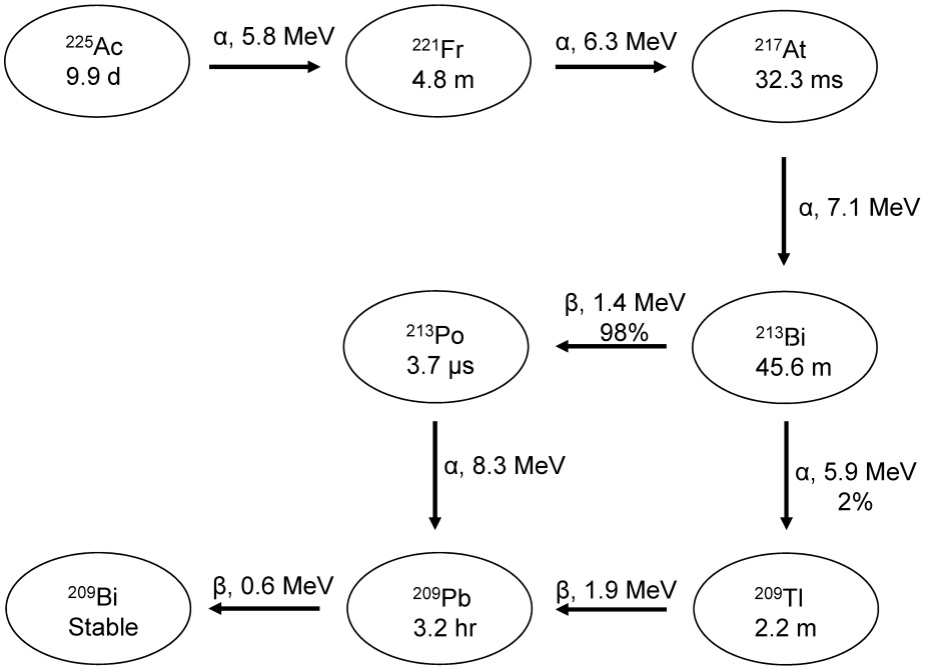
Decay scheme showing the maximum emission energies of ^225^Ac and its daughters.

Some dosimetry issues with ^225^Ac arise regardless of the choice of surrogate. Three of the daughters of ^225^Ac are alpha emitters, shown in Figure 10. Because of the high linear energy transfer (LET) of alphas, the recoil energy of these daughters is over 1000 times larger than the binding energy of typical chemical compounds 173. This causes the daughters to detach themselves from the tracer and freely roam the body and/or lead to radiolysis of the tracer itself. This can increase the absorbed dose to tumors and healthy tissue and needs to be accounted for during dosimetry estimates.

Pharmaceuticals which are internalized and stored in the cell rapidly after binding (such as PSMA-617 88) may suffer less from this effect. Even in this case, a small amount of uncomplexed ^213^Bi (which, when uncomplexed, accumulates in the kidneys 174) may still leave the cell via passive diffusion 175. ^213^Bi was found to be responsible for 60% of the total kidney absorbed dose in mice after injection of [^225^Ac]Ac-huM195 (an anti-CD33 antibody targeting myeloid leukemia cells) 174. The fate of ^221^Fr and astatine-217 (^217^At) *in vivo* is more difficult to determine 176. Research has been done into partially recoil-resistant carriers, however they typically have slower *in vivo* kinetics 177 and may not be ideal for therapy. At this time, the biodistributions of ^225^Ac's daughters are being evaluated using SPECT 178 and *ex vivo* counting of samples, for example using gamma counters 179.

Although we suggested the use of ^177^Lu as a therapeutic nuclide in the previous section, it could uniquely be used for intra-therapy dosimetry of ^225^Ac because they are sometimes administered simultaneously or consecutively in tandem therapies 180. Dosimetry of [^225^Ac]Ac-PSMA-617 using pre-existing [^177^Lu]Lu-PSMA-617 data has been performed in patients, although this has assumed instantaneous decay of ^225^Ac's daughter nuclides 181.

The injected amount of ^177^Lu may be much higher than that of ^225^Ac because of ^225^Ac's potential toxicity; in a tandem therapy published by Rosar et al., mean injected activities of [^225^Ac]Ac- and [^177^Lu]Lu-PSMA-617 were 4 MBq and 6 GBq, respectively 180. Based on approximate SAs of [^225^Ac]Ac- and [^177^Lu]Lu-PSMA-617 88,182, this results in a 5-50 times higher injected mass of ^177^Lu compared to ^225^Ac. Different injected masses may change the biological behavior of the radiopharmaceutical as discussed in Section 4.1.

[^225^Ac]Ac- and [^177^Lu]Lu-DOTATOC were found to have a similar uptake of 7.5 %ID/g in the tumors of nude mice, but there was a 3.8 and 200 times higher uptake of [^225^Ac]Ac-DOTATOC in the kidneys and liver respectively, shown in Table 7. In general, a higher uptake of [^225^Ac]Ac-DOTATOC was found in most organs. Note that the radiochemical purity differed (> 99% and 95% for [^177^Lu]Lu- and [^225^Ac]Ac-DOTATOC respectively) along with the injected mass 183. The higher uptake of [^225^Ac]Ac-DOTATOC means dosimetry with ^177^Lu could severely underestimate the healthy tissue absorbed dose.

The high liver uptake was thought to be due to uncomplexed ^225^Ac in the original sample 183, which has also observed by Miederer et al. 87. The radiochemical purity of [^225^Ac]Ac-PSMA-617 has been quoted as > 98% 88, so biodistribution studies may be more promising for PSMA targeting ligands, however more tests need to be performed. The use of ^225^Ac labeled to DOTA in general has been questioned. The DOTA chelator thermodynamically prefers smaller ions with larger metal centers resulting in less stable complexes, while Ac^3+^ (actinium's most stable oxidation state) is the largest of all known tripositive ions 184. This has been shown to result in demetallation of ^225^Ac from DOTA *in vivo,* which may make high liver uptake difficult to avoid 185.

^89^Zr and ^111^In are both possible surrogates for ^225^Ac, although there has not been enough research to confidently analyze them at this time. However, studies have shown similar biodistributions between ^225^Ac- and ^89^Zr-labeled ofatumumab, an anti-CD20 antibody for NHL 89. ^111^In has been used for pre-clinical dosimetry of ^225^Ac, labeled to hTAB004 (a murine antibody targeting tMUC1) for triple-negative breast cancers 25,91. The biodistributions of [^111^In]In-DTPA-anti-PD-L1-BC and [^225^Ac]Ac-DOTA-anti-PD-L1-BC (an anti-PD-L1 antibody, expressed on various cancers) have been compared in tumor-bearing mice and pharmacokinetic differences in certain tissues were found, with ^111^In having higher %ID/g in the kidneys and tumor, and ^225^Ac having twice as high %ID/g in the liver at the 24 hour timepoint. However, this difference was hypothesized to be due to the differences in catabolism and clearance of the DTPA and DOTA chelates 186.

#### Suitable for theranostic dosimetry?

The recoil of ^225^Ac's daughters make dosimetry challenging, regardless of the choice of surrogate. Current research indicates there may be significant biodistribution differences between ^225^Ac and ^177^Lu labeled to certain pharmaceuticals which could result in absorbed dose underestimation. There has not been enough research into the use of ^89^Zr and ^111^In as surrogates to make conclusions on their suitability. Because of the potential toxicity of ^225^Ac, theranostic dosimetry may be advantageous even if large differences in biodistributions are found, so long as the doses to healthy tissues are not underestimated.

### 5.5 ^64^Cu and ^67^Cu

The isotope pair ^64^Cu and ^67^Cu is a promising option for RPT and theranostics because of copper's versatile coordination chemistry, allowing it to be bound to many small molecules, antibodies and proteins 187. ^67^Cu emits beta particles for therapy and gammas suitable for SPECT imaging, while ^64^Cu can be imaged with PET. ^67^Cu has shown similar therapeutic efficacy to ^177^Lu in mouse studies for prostate cancer 52 and may be more effective than ^131^I for NHL radioimmunotherapy 188.

^67^Cu has been used for intra-therapy dosimetry of itself, such as with BAT-2IT-1A3 (an anti-CD20 antibody for lymphoma) 188. ^64^Cu could be beneficial for pre-therapy dosimetry; [^64^Cu]Cu-Octreotate has been used for theranostic dosimetry of [^67^Cu]Cu-Octreotate in patients 47. Other pharmaceuticals that have been labeled with both^ 67^Cu and ^64^Cu can be found in Table 2.

In certain organs, copper metabolism is independent of SA 54 and therefore biological differences due to different SAs may not be an issue when labeling with some pharmaceuticals such as CuCl_2_. Yumi Sugo et al. 54 found a similar biodistribution of [^64^Cu]CuCl_2_ in tumor-bearing mice using low SA [^67^Cu]CuCl_2_ to that calculated by Qin et al. 55 using high-SA [^64^Cu]CuCl_2_. Similarly, the biodistributions of [^64/67^Cu]Cu-RPS-085 (a PSMA binding ligand) were similar at 4 and 24 hours post-injection when different masses were injected (31 pmol versus 17 pmol respectively) 52 although we note that the difference in injected mass is small.

While there has not been much research comparing ^67^Cu- and ^64^Cu-labeled pharmaceuticals, the research that exists is positive and suggests that ^64^Cu would be suitable for theranostic dosimetry of ^64^Cu, although we assume this pair is still subject to the external factors presented in Section 4.

#### Suitable for theranostic dosimetry?

While there are not many studies comparing the differences between ^67^Cu- and ^64^Cu-labeled pharmaceuticals, the results available at this time are promising and indicate that ^64^Cu could be a suitable imaging surrogate for ^67^Cu.

### 5.6 ^149^Tb, ^152^Tb, ^155^Tb, and ^161^Tb

The terbium isotopes ^152^Tb and ^155^Tb have been proposed as imaging surrogates for the therapeutic ^149^Tb and ^161^Tb. The terbium isotopes are interesting to explore because of their diverse decay characteristics and their high treatment efficacy. Both [^161^Tb]Tb-PSMA-617 and [^149^Tb]Tb-PSMA-617 have been shown to be more effective than [^177^Lu]Lu-PSMA-617 for prostate cancer in mice 189,190, and ^161^Tb delivers higher absorbed doses to water density spheres than ^67^Cu, ^47^Sc, and ^177^Lu in MC simulations 191.

Both of the therapeutic radioisotopes can be imaged for intra-therapy dosimetry. ^161^Tb can be imaged with SPECT which has been done in patients with DOTATOC 192 and ^149^Tb with PET, which has been done in mice with PSMA-617 190. ^161^Tb emits both beta particles and Auger electrons and can be used for dual beta/Auger therapy 61, while ^149^Tb is an alpha emitter. ^149^Tb does not have alpha emitting daughters, which eliminates the potential toxicity from uncomplexed daughters after recoil, such as what occurs with ^225^Ac. However, the daughters of ^149^Tb are long-lived (5+ days), which would need to be considered during dosimetry estimates 193.

The surrogates ^152^Tb and ^155^Tb are suitable for PET and SPECT imaging, respectively. *In vivo* images in mice of each isotope have been obtained with good results 61, and patient PET images have been taken using ^152^Tb-DOTANOC 194 and ^152^Tb-PSMA-617 60.

To our knowledge, biodistribution and pharmacokinetic comparisons between terbium isotopes labeled to identical pharmaceuticals have not been compared, so we cannot comment on the suitability of the terbium isotopes as imaging surrogates. However, we expect they are subject to the same external factors as other same element isotope pairs, discussed in Section 4.

An interesting possibility is the use of ^152^Tb as a PET imaging surrogate for ^177^Lu (in replacement of ^68^Ga). ^152^Tb has a longer half-life (17.5 hours) than ^68^Ga (68 minutes) and more similar chemical properties to ^177^Lu when labeled to PSMA-617 60 because ^177^Lu and ^152^Tb are both lanthanides 195.

#### Suitable for theranostic dosimetry?

More research is needed to identify if ^152^Tb and/or ^155^Tb would be good imaging surrogates for their therapeutic counterparts, ^149^Tb and ^161^Tb. However, we expect they behave similarly to other same-element isotope pairs discussed in this work and would likely be suitable imaging surrogates.

## 6. Discussion

Dosimetry is a crucial step in the personalization of RPT. While the injected activities in patients is usually standardized or scaled by bodyweight 4, it is well known that there is a large variability in radiation sensitivity and pharmacokinetics among patients 5-7. Ignoring this may result in less than optimal treatment outcomes. Although a direct correlation between biological effect and absorbed dose has been difficult to establish in RPT 196, there has been some evidence showing better clinical outcomes with dosimetry calculations 18,21. A study aiming to determine a dose-effect relationship investigated 79 studies and found a correlation between absorbed dose and clinical effect in 48 of them 22. Imaging surrogates could enable personalized dosimetry where it may not otherwise be possible, such as when the therapeutic radionuclide cannot be imaged or can only be imaged with suboptimal image quality.

Many of the therapeutic radionuclides discussed in this work, such as ^131^I, ^177^Lu, ^67^Cu, ^149^Tb, and ^161^Tb can be imaged quantitatively, enabling personalized dosimetry calculations. Intra-therapy dosimetry using the therapeutic radiopharmaceutical should be encouraged whenever possible, as this eliminates most, if not all, of the sources of error discussed in this work. Whether or not to use the therapeutic radiopharmaceutical or an imaging surrogate should be assessed on a case-by-case basis, as the decision depends on many factors, such as the imaging quality, the half-lives of both radionuclides, the pharmaceutical, and the target.

Even when the therapeutic radionuclide can be imaged, an imaging surrogate is desirable for pre-therapy dosimetry. Pre-therapy dosimetry can be especially useful to determine optimal injected activities for the first therapy cycle. An imaging surrogate is also needed for post-therapy dosimetry if the therapeutic radionuclide cannot easily be imaged quantitatively, such as with ^90^Y (besides for radioembolization, in which very high and concentrated activities are injected 150), or ^225^Ac.

When using a surrogate, is it important to consider that there are many factors, extrinsic and intrinsic to the radiopharmaceuticals, that we have discussed in this work that could potentially impact theranostic dosimetry estimates.

The injected mass, SA, and presence of chemical impurities are all external factors which can modify a radiopharmaceutical's biological behavior. Altering any of these variables can potentially change the target-to-background ratio and/or the biodistribution. Most of these effects are partially dependent on the binding site and its saturability, so may be able to be ignored when imaging targets that are not saturable.

Intrinsic to the radiopharmaceuticals, chemical differences between molecules such as the net charge and charge distribution can alter the binding affinity, uptake kinetics, and/or the biodistribution within a given tissue. Post-therapy effects (such as thyroid stunning and the creation of antibodies against the tracer) can impact the binding affinity, the biodistributions, and/or residence times in a given tissue, which is particularly important if only using one scan to perform dosimetry for subsequent therapy cycles. Differences in physical half-lives can cause an inability of the imaging surrogate to capture the washout kinetics of the therapeutic radiopharmaceutical.

If these extrinsic and intrinsic effects become more well understood they may be corrected for, such as through the use of a scaling factor. However, many of these factors are dependent on the pharmaceutical, target site, and/or the radionuclides, so there is no “one-size-fits-all” way of accounting for these differences. Because the biological behavior of a radiopharmaceutical varies on a patient-by-patient basis, a scaling factor would only be entirely accurate if it was also obtained on a patient-by-patient basis, which would be difficult if not impossible to obtain; a population-based scaling factor would increase the accuracy but not be 100% accurate. Some effects like post-therapy effects are harder to account for because they vary heavily among patients 126.

Accounting for differences in physical half-life may not be possible. If the imaging surrogate decays before capturing the therapeutic radiopharmaceutical's washout pharmacokinetics, the true kinetics may never be known (especially if the therapeutic radiopharmaceutical cannot be imaged quantitatively). As stated in Section 4.2, ignoring the long-term slow washout component of the TAC can result in errors in the residence time calculation of up to 45% 29. For this reason, we believe that only radionuclides with physical half-lives comparable to the biological half-lives in the tissue of interest should be used for theranostic dosimetry. The exact cut-off for when the difference in physical half-life is too large is not precisely known and depends on the biological half-life of the radiopharmaceutical in the tissue of interest.

Based on our analysis of specific theranostic pairs, we predict that most same-element isotope pairs are suitable for theranostic dosimetry. It is not guaranteed that the biodistributions and pharmacokinetics of the radiopharmaceuticals of the pair will be identical due to the extrinsic factors we have discussed. However, the extent of these differences is not precisely known. We believe, however, that the potential benefits of personalized dosimetry outweigh the differences. An exception to this, as stated, is if the physical half-life of the imaging surrogate is too short, such as with ^123^I and ^131^I.

Many different-element pairs appear to have significant intrinsic differences that may result in over/underestimation of absorbed dose, such as difference in bone marrow uptake of ^111^In versus ^90^Y 99. However, it is not a lost cause for different-element pairs; as mentioned if the differences between the radionuclides become known more precisely for different pharmaceuticals and treatments, scaling factors may be used to improve dosimetry estimates. Again, we believe an exception to this may be if the differences in physical half-lives are too large.

Something that is important to address is the extent to which the issues discussed in this work impact dosimetry calculations. As mentioned previously, personalized dose assessments, even under optimal conditions, have errors that can be higher than 20% 103. If the biodistribution in the tissue of interest differs by a few percent between the imaging and therapeutic radiopharmaceutical, the addition of error to the absorbed dose estimate may be small compared to pre-existing errors and therefore unlikely to cause a big impact.

Furthermore, even if there are significant differences in biological behavior, another question, perhaps the most important, should be considered: does dosimetry with an imaging surrogate result in better patient outcomes compared to not performing dosimetry at all, or compared to performing dosimetry only with the therapeutic radiopharmaceutical? If pre-therapy dosimetry with an imaging surrogate results in better patient outcomes, it should still be performed even if large biological differences between the surrogate and the therapeutic radiopharmaceutical are discovered, so long as there is not the potential for toxicity if the absorbed doses are underestimated.

To this end, an optimal procedure may be to perform pre-therapy dosimetry with an imaging surrogate, and intra-therapy dosimetry with the therapeutic radiopharmaceutical (if it can be imaged) after each cycle to optimize injected activities in subsequent cycles. For therapeutic radiopharmaceuticals which cannot be imaged, performing dosimetry with an imaging surrogate post-therapy should be also encouraged if it results in better patient outcomes.

At this time, personalized dosimetry is not routinely performed. This is vastly different from external beam radiotherapy (EBRT), in which every patient is given a detailed treatment plan to guide their therapy. It would be unthinkable in EBRT to administer therapy to a patient without a thorough treatment plan. Nonetheless, individual treatment plans are not common in RPT. They require multiple SPECT/CT or PET/CT scans, which are limited by cost, resources, and difficulty. Furthermore, for many patients, returning to the clinic for up to 4 scans after each cycle of therapy can be a large burden. In these cases, the initial diagnostic scan may be all that is available. The dose-response relationship is also significantly different for RPT than EBRT, for reasons such as heterogeneous dose distributions (due to varying tracer diffusion efficacy in the tumor tissue and/or cellular uptake, instead of being able to guide the beam in EBRT) and the much lower absorbed dose rate in RPT 197.

While research is ongoing, there are ways to reduce some of the burdens associated with dosimetry. Single time point dosimetry may allow accurate dosimetry estimates with less SPECT/CT and PET/CT scans. If complete dosimetry data (3 time points) are obtained after the first cycle of therapy, using a single time point at 24 hours after subsequent therapeutic injections only leads to a 2% difference in renal absorbed dose compared to taking a full data set after each cycle 13. There is also commercial software available which can make dosimetry more user-friendly to clinics, such as the Dosimetry Toolkit 14 and OLINDA/EXM 15 with more manufacturers and software companies entering the market.

There has not been much research into the clinical impact of dosimetry with an imaging surrogate compared to dosimetry with the therapeutic radionuclide only. However, more work needs to be done to increase the prevalence of personalized dosimetry in general, and one way to do that is through theranostic dosimetry. As stated by Michael Stabin, “treating all nuclear medicine patients with a single, uniform method of activity administration amounts to consciously choosing that these patients be treated with a lower standard of care than patients who receive radiation externally for cancer treatments.” 198.

To this end, it is important to correlate theranostic and non-theranostic dosimetry with patient outcomes to ensure the highest possible standard of care and to determine the impact of the extrinsic and intrinsic factors we have discussed here on actual clinical and preclinical studies. We believe if care is taken to consider differences in biological behavior and other aspects discussed in this work between radiopharmaceuticals labeled with different pairs of isotopes, theranostic dosimetry would be immensely beneficial for patient care.

## 7. Conclusion

This review was an attempt to answer the question: is it possible to accurately determine absorbed dose estimates for a therapeutic radiopharmaceutical by extrapolation from its imaging counterpart? There can be large differences in the biological behavior between identical pharmaceuticals labeled with different radioisotopes or radionuclides. Variables such as the injected amount/mass, physical-half-life, coordination chemistry, specific activity of the radiopharmaceutical, presence of chemical impurities, and patient behavior can all alter the biodistribution and pharmacokinetics of a radiopharmaceutical. These differences may potentially cause errors in absorbed dose estimates when using one radiopharmaceutical to predict the absorbed dose deposited by another radiopharmaceutical, even if the isotopes are of the same chemical element. Based on existing studies, same-element isotope pairs seem to be suitable for theranostic dosimetry; the benefits of dosimetry outweigh the differences in the biological behavior except when the physical half-life of the imaging surrogate is very short. The behavioral differences between many different-element pairs may be too large and result in significant errors in absorbed dose estimates, which could lead to over- or under-treatment. More research needs to be done analyzing the impact of these differences on dosimetry on a case-by-case basis for different radiopharmaceutical pairs. More efforts also need to be devoted to correlating patient outcomes with theranostic dosimetry, to determine if performing dosimetry with an imaging surrogate improves outcomes even if there are large behavioral differences between the radiopharmaceutical pairs.

## Figures and Tables

**Table 1:** Decay information for popular radionuclides used as theranostic pairs 39

Nuclide	Half-Life (hours)	Decay Mode	Possible Imaging Modality	γ Rays		Max Particulate Energy (keV)			
				Prominent E_γ_ (keV)	Intensity (%)	E_β+_	E_β-_	E_α_	E_Auger_
**Imaging Nuclides**									
^86^Y	14.7	β^+^ (32)	PET	443	17	1481	--	--	--
		EC (67)		627	33				
^64^Cu	12.7	β^+^ (18)	PET	--	--	653	579	--	--
		EC (44)							
		β^-^ (38)							
^123^I	13.2	EC (100)	SPECT	159	83	--	--	--	23
^124^I	101.0	β^+^ (23)	PET	602	63	2138	--	--	23
		EC (77)							
^43^Sc	3.9	β^+^ (88)	PET	373	23	1199	--	--	--
		EC (12)							
^44^Sc	4.0	β^+^ (94)	PET	--	--	1474	--	--	--
		EC (6)							
^83^Sr	32.4	β^+^ (26)	PET	381	14	1251	--	--	--
		EC (74)							
^85^Sr	1555.0	EC (100)	SPECT	514	96	--	--	--	--
^152^Tb	17.5	β^+^ (20)	PET	344	64	1720	--	--	35
		EC (80)							
^155^Tb	127.0	EC (100)	SPECT	87	32	--	--	--	--
				105	25				
^203^Pb	51.9	EC (100)	SPECT	279	81	--	--	--	55
^68^Ga	1.1	β^+^ (89)	PET	--	--	1899	--	--	--
		EC (11)							
^18^F	1.8	β^+^ (97)	PET	--	--	633	--	--	--
		EC (3)							
^111^In	67.2	EC (100)	SPECT	171	91	--	--	--	19
				245	94				
^99m^Tc	6.0	IT (100)	SPECT	140	89	--	--	--	16
^89^Zr	78.4	β^+^ (23)	PET	909	99	902	--	--	13
		EC (77)							
							
**Therapeutic Nuclides**									
^90^Y	2.7	β^-^ (100)	Brems., PET	--	--	--	2280	--	--
^67^Cu	2.5	β^-^ (100)	SPECT	93	16	--	562	--	
				185	49				
^125^I	59.4	EC (100)	SPECT	3	15	--	--	--	23
				27	113				
				30	13				
^131^I	8.02	β^-^ (100)	SPECT	364	82	--	807	--	25
^47^Sc	3.3	β^-^ (100)	SPECT	159	68	--	600	--	--
^89^Sr	50.2	β^-^ (100)	--	--	--	--	1501	--	--
^149^Tb	0.17	β^+^ (7)	PET, SPECT	165	26	2450	--	3967	35
		EC (76)		352	29				
		α (17)		388	18				
				652	16				
^161^Tb	6.9	β^-^ (100)	Brems.	75	10	--	593	--	37
^212^Pb	0.44	β^-^ (100)	SPECT	239	44	--	570	8736	58
^177^Lu	6.6	β^-^ (100)	SPECT	208	10	--	497	--	--
^225^Ac	10.0	α (100)	SPECT	218	11	--	--	5830	--
				440	26				

EC = electron capture, IT = isomeric transition. Prominent gammas are defined as those with intensity greater than 10%. Energies for Auger electrons with intensities greater than 1% were included. Brems. = Bremsstrahlung imaging (SPECT).

**Table 2:** Popular same-element pairs and common targets, pharmaceuticals, and treated diseases

Nuclides		Targets	Pharmaceuticals	Applications	References
Therapeutic	Imaging				
^90^Y	^86^Y (PET)	Somatostatin Receptors (SSTRs)	Somatostatin Analogues, e.g. DOTATOC	NETs	34
		CD20 Antigens	mAbs, e.g. Ibritumomab Tiuxetan	Non-Hodgkin's Lymphoma (NHL)	23
		HER2/neu	Trastuzumab	Ovarian Cancer	40
		Prostate Specific Membrane Antigens (PSMA)	PSMA 4-6	Prostate Cancer	41
^131^I	^123^I (SPECT)/^124^I (PET)	Thyroid-Stimulating Hormone (TSH)	Sodium Iodide (NaI)	Thyroid Diseases	42
		Norepinephrine Transporters	mIBG	Neuroblastomas, Pheochromocytomas, etc.	35,43,44
		PSMA	MIP-1095	Prostate Cancer	45
		The Extra Domain B of Fibronectin	L19-SIP	Multiple Cancers (Melanoma, Head/Neck, etc.)	46
^67^Cu	^64^Cu (PET)	SSTRs	Octreotate, SarTATE	NETs, Neuroblastomas	47,48
		CD20 Antigens	2IT-BAT-Lym-1	NHL	49
		PSMA	PSMA-617, RPS-085	Prostate Cancer	50-52
		Copper Transporter Protein 1	Cl_2_	Multiple Cancers (Colorectal, Breast, Prostate, Melanoma, etc.)	53-55
^47^Sc	^89^Sr* (PET)/^44^S (PET)	SSTRs	DOTANOC (A Somatostatin Analogue)	NETs	56
		Folate Receptors	cm10 (a DOTA-Folate Conjugate)	Multiple Cancers (Breast, Ovarian, Lung, etc.)	57
^89^Sr	^83^Sr* (PET)	Bone	Chloride (Cl)	Bone Metastases	58
^161^Tb/^149^Tb	^152^Tb (PET)/^155^Tb (SPECT)	SSTRs	DOTANOC	NETs	59
		PSMA	PSMA-617	Prostate Cancer	60
		Folate Receptors	cm09	Multiple Cancers (Breast, Ovarian, Lung, etc.)	61

*We list these pairs here for completeness, but they are not discussed further in this work.

**Table 3:** Popular different-element pairs and the common targets, pharmaceuticals, and treated diseases

Nuclides		Targets	Pharmaceuticals	Applications	Reference
Therapeutic	Imaging				
^177^Lu	^68^Ga (PET)	SSTRs	DOTATATE, DOTATOC	NETs	62,63
		PSMA	PSMA-617/PSMA-11, PSMA-I&T	Prostate Cancer, Glioblastomas	64,65
		Gastrin-Releasing Peptide Receptor (GRPR)	NeoBOMB1, RM2, AMBA	Multiple Cancers (Prostate, Breast, etc.)	66-68
		Bone	DOTA^ZOL^	Bone Metastases	69,70
	^89^Zr (PET)	Epidermal Growth Factor Receptor (EGFR)	Cetuximab	Multiple Cancers (Colorectal, Head/Neck, Skin)	71
		CD38 Antigens	Daratumumab	Lymphoma	72
		Glypican-1	Miltuximab	Prostate Cancer	73
		PD-L1	αPD-L1, Y003	Colon Carcinoma	74
	^64^Cu (PET)	SSTRs	DOTATOC, DOTATATE	NETs	37,75
		PSMA	PSMA-617	Prostate Cancer	76,77
		Vascular EGFR	diZD	Breast Cancer	78
		L1-CAM	chCE7 fragments, cA10-A3	Multiple Cancers (Neuroblastomas, Ovarian, Endometrial, etc.)	79,80
		EGRF	Cetuximab	Esophageal Squamous Cell Carcinoma	81,82
	^18^F (PET)	PSMA	PSMA-617	Prostate Cancer	77
	^44^Sc (PET)	PSMA	PSMA-617	Prostate Cancer	83-85
^225^Ac	^177^Lu (SPECT)	SSTRs	DOTATATE, DOTATOC	NETs	86,87
		PSMA	PSMA-617	Prostate Cancer	88
	^89^Zr (PET)	CD20 Antigens	Ofatumumab	NHL	89
		EGRF	Nimotuzumab	Multiple Cancers (Squamous Cell Head/Neck, Breast, Cervical, etc.)	90
	^111^In (SPECT)	Tumor-Associated-MUC1	TAB004	Breast Cancer	25,91
^90^Y	^177^Lu* (SPECT)	SSTRs	DOTATATE, DOTATOC	NETs	92
		PSMA	PSMA-617, J591	Prostate Cancer	93,94
	^111^In (SPECT)	SSTRs	DOTATATE, DOTATOC	NETs	95,96
		CD20 Antigens	Ibritumomab Tiuxetan	NHL	97
		PSMA	J591	Prostate Cancer	94,98
		IL-2Rα	Anti-Tac	T-Cell Leukemia	99
		Lewis^Y^ Carbohydrate Antigen	mAbs, e.g. B3	Multiple Cancers (Colorectal, Breast, Esophageal, etc.)	100
	^99m^Tc (SPECT)	--	[^99m^Tc]Tc-MAA with ^90^Y Microspheres	Hepatocellular Carcinoma, Metastatic Liver Cancers	101,102
	^89^Zr* (PET)	CD20 Antigens	Ibritumomab Tiuxetan	NHL	24

*We list these pairs here for completeness, but they are not discussed further in this work.

**Table 4:** The impact of therapy with [^90^Y]Y-DOTATOC on the residence time of [^111^In]In-octreotide and the estimated BED from [^90^Y]Y-DOTATOC (adapted from Binnebeek et al. 96)

Organ	Change in Residence Time Pre vs. Post-Therapy (%)	Change in BED Pre vs. Post-Therapy (%)
Red Marrow	347	155
Spleen	126	--
Kidneys	80	85
Liver	76	--
Remainder	76	--

**Table 5:** The theranostic pairs discussed in the following sections and how the physical half-lives of the imaging surrogates compare to their therapeutic counterparts

Therapeutic Radionuclide	Imaging Surrogate	Percentage of Therapeutic Counterparts Half-Life (%)
^131^I	^123^I	6.9*
	^124^I	52.5
^90^Y	^99m^Tc	9.3*
	^111^In	103.7
	^86^Y	22.7
^177^Lu	^68^Ga	0.69*
	^44^Sc	2.5*
	^64^Cu	8.0*
	^18^F	1.1*
	^89^Zr	49.3
^225^Ac	^177^Lu	66.9
	^89^Zr	32.9
	^111^In	28.2
^67^Cu	^64^Cu	21.2
^149^Tb	^152^Tb	428.9
	^155^Tb	3112.7
^161^Tb	^152^Tb	10.6
	^155^Tb	76.7

*These surrogates have the most significant differences, with physical half-lives of less than 10% of their therapeutic counterparts.

**Table 6:** Correlation strength between ^68^Ga PET and ^177^Lu SPECT markers, using Spearman rank correlation analysis, when used to label different pharmaceuticals. AUC = area under the curve, D/A_0_ = absorbed dose per injected activity

^68^Ga PET Marker	^177^Lu SPECT Marker	Tracer	Location	Correlation	Source
SUV_mean_	Effective Dose	PSMA-617	Main Organs	Moderate (r = 0.61, P < 0.001)	Wang et al. 64
SUV_mean_	AUC	PSMA-617	Tumor Lesions	High (r = 0.907, P < 0.001)	Wang et al. 64
SUV_max_	AUC	PSMA-617	Tumor Lesions	High (r = 0.915, P < 0.001)	Wang et al. 64
SUV_mean_	D/A_0_	DOTATOC (^68^Ga) & Octreotide (^177^Lu)	Tumor Lesions	Moderate (r = 0.72, P < 0.001)	Ezziddin et al. 62
SUV_max_	D/A_0_	DOTATOC (^68^Ga) & Octreotide (^177^Lu)	Tumor Lesions	Moderate (r = 0.71, P < 0.001)	Ezziddin et al. 62
SUV_mean_ (in lesions)	Dose	PSMA-11 (^68^Ga) & PSMA-617 (^177^Lu)	Whole Body	Moderate (r = 0.62, P <0.01)	Violet et al. 38
SUV_max_ (before therapy)	Maximum Voxel Dose	DOTATOC/DOTATATE	Tumor Lesions	Moderate (r = 0.76, P = 0.02)	Hänscheid et al. 63
SUV_max_ (after therapy)	Maximum Voxel Dose	DOTATOC/DOTATAT	Tumor Lesions	High (r = 0.99, P = 0.003)	Hänscheid et al. 63

**Table 7:** Uptake values in %ID/g of [^225^Ac]Ac- and [^177^Lu]Lu-DOTATOC in tumor-bearing mice as reported by Graf et al. 183

Organ	[^225^Ac]Ac-DOTATOC (%ID/g)	[^177^Lu]Lu-DOTATOC (%ID/g)	% Difference
Tumor	7.5	7.5	0
Kidneys	5.8	1.5	3.87
Liver	> 10	0.05	200
Heart & Muscle	< 1	< 1	0

## Abbreviations

%ID/g: percentage injected dose per gram
AUC: area under the curve
BED: biological effective dose
CT: computed tomography
D/A_0_: absorbed dose per injected activity
DFO: deferoxamine
EBRT: external beam radiotherapy
EC: electron capture
EGFR: epidermal growth factor receptor
FDG: fluorodeoxyglucose
GRPR: gastrin-releasing peptide receptor
IT: isomeric transition
LET: linear energy transfer
mAb: monoclonal antibody
MC: Monte Carlo
mIBG: metaiodobenzylguanidine
MIRD: Medical Internal Radiation Dose
NaF: sodium fluoride
NaI: sodium iodide
NETs: neuroendocrine tumors
NHL: non-Hodgkin's lymphoma
PET: positron emission tomography
PSMA: prostate specific membrane antigen
ROI: region of interest
RPT: radiopharmaceutical therapy
SA: specific activity
SPECT: single photon emission computed tomography
SSTRs: somatostatin receptors
SUV: standard uptake values
TAC: time activity curve
TIA: time integrated activity
TSH: thyroid-stimulating hormone
VOI: volume of interest
WBS: whole-body scintigraphy
